# Islands of Biodiversity: Characterization of Lichen Flora in Antarctic Nunataks

**DOI:** 10.3390/jof12050314

**Published:** 2026-04-24

**Authors:** Ana Aramburu, Núria Beltran-Sanz, José Raggio, Pradeep K. Divakar, Ana Pintado, Asunción de los Ríos, Leopoldo G. Sancho

**Affiliations:** 1 Department of Pharmacology, Pharmacognosy and Botany, Faculty of Pharmacy, Complutense University of Madrid, Plaza Ramón y Cajal s/n, 28040 Madrid, Spain; beltrann@natur.cuni.cz (N.B.-S.); jraggioq@ucm.es (J.R.); pdivakar@ucm.es (P.K.D.); apintado@ucm.es (A.P.); sancholg@ucm.es (L.G.S.); 2 Department of Botany, Charles University, Benátská 2, Praha 2, 12800 Prague, Czech Republic; 3 Department of Biogeochemistry and Microbial Ecology, National Museum of Natural Sciences (MNCN-CSIC), c/Serrano 115 dpdo, 28006 Madrid, Spain; arios@mncn.csic.es

**Keywords:** Antarctica, biogeography, cryptogams, DNA barcoding, lichen-forming fungi, species diversity, vegetation survey

## Abstract

Antarctic terrestrial photosynthetic biota is dominated by cryptogamic communities, which are largely restricted to scarce ice-free areas. Among these, nunataks constitute habitats of remarkable biogeographical interest, as they may harbor distinctive biotic assemblages worthy of investigation. This work presents a comprehensive assessment of lichen diversity on Antarctic nunataks. The lichen flora of four nunataks on the Hurd Peninsula (Livingston Island, maritime Antarctica) was investigated. Specimens were identified using an integrative approach combining morphological assessment and DNA barcoding. This survey revealed a high and potentially underestimated species richness, with 39 confidently identified and several additional taxa requiring further taxonomic resolution. A review of published records of lichen occurrence in nunatak and non-nunatak environments throughout Antarctica was used to evaluate patterns in taxonomic, biogeographical, and morphotype composition. This synthesis showed that nunataks support lower species richness than other ice-free environments. Most of their taxa occur in non-nunatak areas, consistent with patterns observed locally on the Hurd Peninsula. Floristic overlap seems greater in continental Antarctica, suggesting a stronger influence of nunatak-associated environmental constraints in the maritime region. These results underscore the ecological significance of nunataks as environmentally filtered habitats and highlight their relevance for understanding biodiversity patterns and community assembly in Antarctica’s terrestrial ecosystems.

## 1. Introduction

Antarctica constitutes a unique biome, as it is the only continent whose terrestrial photosynthetic biota is dominated by a multi-organism cryptogamic cover composed primarily of lichens and bryophytes, together with free-living algae and cyanobacteria [1]. Terrestrial vegetation is restricted to a minute fraction of fragmented, permanently ice-free terrain, estimated to comprise roughly 0.18% [2] to about 0.44% [3] of the continent’s 14 million km^2^ area [4,5]. Exposed ground occurs mainly at low elevations in coastal regions, along glacial margins, and on exposed mountain ranges. Among the few terrestrial habitats devoid of permanent snow and ice—consisting mostly of bare soil and rock—rock surfaces in particular exhibit remarkable bioreceptivity. Owing to their stability and durability, they serve as the primary, and oftentimes the only, substrate available for colonization by Antarctic cryptogamic tundra communities [6].

Vegetation is predominantly concentrated along the coast of the northern maritime Antarctic phytogeographic zone, especially on the northwestern side of the Antarctic Peninsula (up to approximately 68° S) and across the South Shetland and South Orkney archipelagos [5,7,8,9,10]. Although spatially extensive bryophyte and lichen carpets may develop near the shoreline and in other favorable microsites, vegetation cover generally remains sparse and discontinuous, forming patchy assemblages characteristic of fellfield habitats [6,11]. The relatively mild and moist maritime climate of the northwestern Antarctic Peninsula and its associated offshore islands provides environmental conditions sufficiently favorable to support the richest flora and highest vegetation density of all Antarctic biogeographic regions [7]. This area also harbors the continent’s only two native flowering plants: the Antarctic hairgrass Deschampsia antarctica É.Desv. and the Antarctic pearlwort *Colobanthus quitensis* (Kunth) Bartl. [8].

The pronounced patchiness of Antarctic vegetation is mirrored by the current state of knowledge of terrestrial biodiversity across the continent, which remains uneven and incomplete [12]. This unevenness is evident not only among the 16 biologically distinct Antarctic Conservation Biogeographic Region (ACBRs; [9]), partly as a consequence of accessibility constraints and occasional sampling bias towards areas nearing research stations [13], but also along a marked coastal-inland gradient: 77.8% of terrestrial biodiversity records from ice-free areas occur within less than 1 km of the coastline, with record density declining progressively with increasing distance inland [12]. Across multiple taxonomic groups, coastal areas are generally regarded as more diverse than inland ice-free sites [14]. In the case of vegetation, and along the Antarctic Peninsula in particular, both diversity and abundance have been shown to consistently decrease southwards and with increasing distance from the sea [5,10]. Nevertheless, despite these well-documented regional patterns, cryptogamic floras inhabiting high-elevation inland environments across Antarctica remain comparatively underexplored and poorly characterized relative to those of coastal sites.

In this regard, nunataks—derived from the Inuit word *nunataq*, meaning “lonely peak” [15,16]—are of particular interest. The term refers to deglaciated mountain summits, ridges, and bedrock promontories that rise above the surface of surrounding glaciers, ice sheets, or snowfields. Encircled by permanent ice, nunataks are relatively isolated from other ice-free substrata and decoupled, to varying degrees, from the buffering climatic influence of the ocean, partly depending on their geographic position [17]. This isolation renders nunataks distinctive terrestrial habitats within the Antarctic landscape.

Studies of lichen assemblages on nunataks from both western and eastern continental Antarctica have consistently shown that these environments support lower taxonomic diversity than nearby coastal areas. Examples include Northern Victoria Land, where lichen diversity declines from a coastal ridge to a ca. 750 m high mountain located approximately 70 km inland [18]; Mac. Robertson Land, where the poorly populated Depot Peak, about 160 km farther inland, contrasts with the more diverse rocky outcrops along the coast and adjacent inland features [19]; and the Prydz Bay area, where a group of inland nunataks part of the Prince Charles Mountains are notably less diverse than the coastal oases of the Larsemann and Vestfold Hills [20]. Overall, these patterns support the view that marked differences in the extent, continuity, and connectivity of ice-free habitats across landscape types play a key role in shaping Antarctic floristic patterns. Taxonomic richness tends to be highest at low elevations along the coastal fringe, where suitable substrates are generally more extensive, diverse, and spatially continuous [5], whereas surfaces available for colonization on nunataks are comparatively limited and highly fragmented.

Multiple underlying environmental drivers likely contribute to the reduced diversity at higher, more inland sites compared with neighboring coastal lowlands. Said drivers encompass lower temperatures [21], greater exposure to prevailing winds and associated mechanical abrasion by wind-blown rock debris or ice crystals [22,23], and water shortage resulting from enhanced desiccation, which in turn is driven by strong wind exposure, as well as by differences in precipitation regimes between landscape types, which shape the relative importance of snowfall, drifting snow, condensation, and meltwater as hydration sources [11,23]. Elevated ultraviolet radiation and higher overall insolation—partly due to light reflection from surrounding snow [24]—further intensify environmental severity in nunatak settings. Ecological constraints linked to the remoteness of these rocky outcrops from nearby ice-free biological source areas, together with the insulating effect of the surrounding glaciers [25], also restrict colonization and consequently reduce floristic richness. These limitations manifest as reduced nutrient input resulting from the absence of nearby seabird nesting sites, penguin rookeries, and seal colonies [26,27,28,29,30,31,32,33,34], minimal mineral- and nitrogen-rich underglacial water drainage [35], lower deposition of marine aerosols [36,37], and a relatively minor impact of trampling by penguin populations and human visitors. In fact, nutrient-availability gradients have been described in the South Bay area (Livingston Island, South Shetland Islands), ranging from enriched coastal sites at low elevations near penguin rookeries and exposed to milder temperatures to comparatively nutrient-deficient habitats at higher elevations farther inland [38]. In the Robertskollen nunatak group in western Dronning Maud Land, differences in soil nutrient concentration arise between sites adjacent to breeding colonies of flying seabirds and sites with a lower affluence of ornithogenic products [17,39,40,41], which in turn translates into differences in vegetation composition and cover [42]. The latter is higher around enriched sites, which hints at lichen growth being nutrient-limited at inland Antarctic mountains [42]. Given their distinctive combination of substrate limitation, climatic severity, and restrictive ecological conditions, nunataks represent sites of notable biogeographical interest with the potential to harbor unique biotic assemblages, making their floristic composition particularly worthy of investigation.

Against this substantially continental backdrop, it remains unclear how nunataks situated within floristically rich regions of maritime Antarctica compare to their coastal counterparts. The Hurd Peninsula in particular (Livingston Island, South Shetland Islands) is an area of remarkable floristic significance. Ice-free surfaces on the peninsula, covering approximately 3 km^2^, are distributed across bays, raised beaches, cliffs, scree slopes, meltwater channels, and glacial moraines surrounding South Bay [43,44]. These predominantly coastal sites have been the focus of intensive botanical surveys over recent decades [21,43,44,45,46], and the area is now recognized as harboring an exceptionally abundant and diverse cryptogamic flora. To date, 196 lichen taxa [44] and 46 bryophyte taxa [45] have been recorded, representing approximately 40% of known Antarctic lichen diversity (based on the 484 lichenized fungal taxa reported by Øvstedal and Lewis Smith [47]) and about 34% of Antarctic moss diversity. Conversely, mountainous sites farther inland remain insufficiently characterized. Thus far, only the lichen flora of Moores Peak and the lithic and composite soil prokaryotic communities of both MacGregor Peak and Moores Peak have been examined [44,48]. Given the evident paucity of floristic surveys on nunataks in the region, fundamental questions persist regarding the taxonomic composition, species richness, and biogeographical affinities of the lichen communities supported by these underrepresented habitats. Previous surveys have shown that lichen assemblages at Moores Peak are less diverse than those occurring at more coastal sites [44], consistent with observed declines in species richness along altitudinal and inland gradients in the South Bay area, from the shoreline to higher interior locations such as Reina Sofia Peak (274 m) [21,43]. Comparable trends have also been reported from Admiralty Bay in King George Island [49].

The primary aim of the present study is to provide a comprehensive evaluation of lichen biodiversity on Antarctic nunataks. We present new field-based taxonomic records from four previously unexplored nunataks on the Hurd Peninsula, identified through an integrative approach combining morphological assessment with conventional DNA barcoding techniques. In doing so, we seek to enhance current understanding of the diversity and distribution of nunatak lichen communities within the maritime Antarctic region. Beyond providing a local inventory of lichenized fungi, we conducted an exhaustive compilation and critical review of the published literature on lichens occurring in nunatak environments across Antarctica. This synthesis allowed us to examine nunatak lichen floras in terms of taxonomic diversity, biogeographical distribution, and morphotype composition. Overall, by integrating new empirical data with a continent-wide literature synthesis, the study aims to elucidate the ecological role of nunataks relative to neighboring deglaciated areas across the contrasting climatic regimes of maritime and continental Antarctica, and to shed light on how geographic isolation and environmental stress shape community assembly in these still poorly characterized habitats.

## 2. Materials and Methods

### 2.1. Study Area and Sampling Strategy

**Figure 1:**
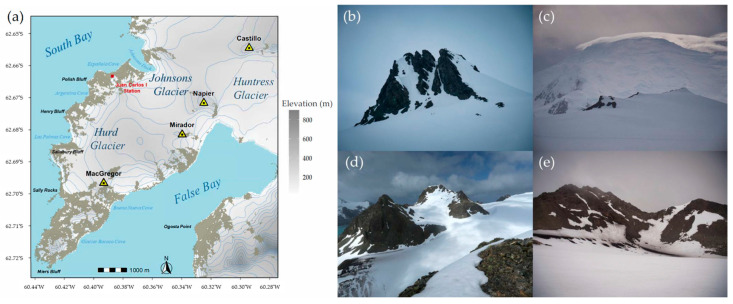
(**a**) Location of the study sites within the Hurd Peninsula. DEM derived from the REMA, courtesy of the Polar Geospatial Center. Data on the Antarctic coastline and rock outcrop sourced from the SCAR Antarctic Digital Database. Light-brown areas indicate snow- and ice-free substrates. Grey shading represents elevation, with shading intensity proportional to altitude. Blue contour lines at 50 m intervals. Surveyed nunataks are shown as yellow triangles. (**b**) Northwestern side of Nunatak del Castillo; (**c**) Overview of the outcrop series forming the Napier Peak complex. (**d**) Northern slopes of Cerro Mirador, viewed from Napier Peak. (**e**) Western-facing slopes of MacGregor Peak. Photographs by Leopoldo G. Sancho (**d**) and Sergi Ricart Ibars (**b**,**c**,**e**).

Livingston Island, the second largest island of the South Shetland archipelago after King George Island, with a surface area of approximately 798 km^2^, is located between 62°27′32″–62°45′29″ S and 59°48′21″–61°10′57.3″ W, about 800 km south-southeast of Cape Horn and 110 km northwest of Cape Roquemaurel, at the tip of the Antarctic Peninsula [50]. The present study focuses on the Hurd Peninsula, located at the southern end of Livingston Island and bordered by South Bay to the north and False Bay to the south (Figure 1a). The area is predominantly influenced by warm and moist air masses from the southeast Pacific. To the southwest, the bordering Friesland Ridge (1700 m) provides shelter from the less frequent cold and dry southerly winds [51]. As a result, the region experiences less pronounced daily and seasonal thermal fluctuations, frequent precipitation—mainly in the form of rain—and a persistent, dense cloud cover favored by the neighboring mountain ranges, which limits direct solar radiation [52,53,54]. In contrast to the rest of Livingston Island and much of the South Shetland archipelago which are primarily volcanic, the Hurd Peninsula is composed of an acidic sedimentary bedrock originating from the Miers Bluff Formation [55].

To create the map of the Hurd Peninsula, we combined version 2 mosaic tiles of the Reference Elevation Model of Antarctica (REMA) at a 32 m spatial resolution [56] with high-resolution vector polygons of the Antarctic coastline [57] and the automatically extracted rock outcrop dataset for Antarctica [58], both sourced from the SCAR Antarctic Digital Database. The map was visualized using the *sf* [59], *terra* [60], and *ggplot2* [61] R packages in RStudio v2026.01.0+392 (Posit Software, Boston, MA, USA).

The study area comprises four nunataks (Nunatak del Castillo, Napier Peak, Cerro Mirador, and MacGregor Peak) protruding through the ice cap that extends between the Huntress Glacier to the east, the Johnsons Glacier to the northeast, and the Hurd Glacier to the southwest (Figure 1a–e and Figure S1).

Sampling was carried out during the Spanish Antarctic Campaigns of 2021–2022 and 2022–2023 (Appendix A). Nunatak del Castillo, the northernmost and most inland site, has been proposed as the remnant of a volcanic structure [62]. The other three nunataks are situated closer to the coastline, overlooking False Bay, and are therefore likely subject to a greater degree of oceanic influence in comparison. Their seaward sides (with a south-southeastern orientation) usually corresponded to their most precipitous walls, which rendered them inaccessible. Nunatak del Castillo was surveyed on more occasions than the other three sites (Appendix A) owing to its prominence, greater geographical isolation, and comparatively high microtopographical complexity. Despite logistical constraints imposed by limited terrain accessibility, sampling at all four nunataks was nonetheless meticulously conducted across a diverse range of orientations, slopes, and elevations. Surveys were opportunistic rather than spatially explicit [43,45,63]. No fixed spatial separation was imposed between potentially conspecific sampled specimens. A total of 280 specimens were collected from exposed rock surfaces free of snow and ice and, when available, from newly formed soils typically found on rock crevices. To avoid oversampling, collection was restricted to what was strictly necessary, with priority given to taxa lacking distinctive macroscopic features—mainly inconspicuous thalli closely attached to the substratum—and limiting repeated sampling of easily identifiable, charismatic specimens. This approach helped protect local flora and minimize the ecological impact of the study. Immediately after collection, samples were transported to the laboratory facilities of the Juan Carlos I Antarctic Base, allowed to air-dry completely at room temperature (approximately 15 °C), stored at −20 °C, and transported to the Complutense University of Madrid, keeping them frozen uninterruptedly until analysis was resumed.

### 2.2. Taxonomic Identification of Lichen Samples

#### 2.2.1. Phenotypical Analysis

Qualitative phenotypic traits of all collected samples were analyzed. Morphological characters such as thallus type, color, surface texture, and features of reproductive structures (when present) of all collected specimens were examined under a Nikon SMZ445 stereo microscope (Nikon Corporation, Tokyo, Japan). Anatomical structures, including the organization of tissue layers, the arrangement and morphology of asci (when present), and ascospore characteristics, were studied under a Nikon H550S microscope (Nikon Corporation, Tokyo, Japan) using thin, hand-cut sections mounted in water. Specimens were identified based on the available literature describing the lichen flora of the area and adjacent islands, including Olech [64], Øvstedal & Lewis Smith [8], and Søchting et al. [44]. To further assist with identification, spot tests were used for chemical examination of selected groups, following the methodology of Orange et al. [65]. When specimens exhibited close morphological similarity to a known species but showed some degree of distinction from certain typical features, the abbreviation aff. (*affinis*) was used to denote this uncertainty. Specimens that clearly belonged to a particular genus based on phenotypic traits but lacked sufficient development of diagnostic structures for confident species-level assignment were designated only at the genus level [66].

#### 2.2.2. Molecular Analysis

##### DNA Extraction, Amplification, and Sequencing

Out of the collected specimens, 102 that had been tentatively identified to species or genus level underwent molecular analyses to confirm, correct or refine the original classification. To minimize pseudoreplication associated with the sequencing of potentially clonal material, only one morphologically unidentifiable thallus per rock fragment was selected for DNA barcoding when multiple similar thalli were present. Fragments of vegetative or reproductive material showing no visible signs of parasitism by lichenicolous fungi were selected for DNA extraction. Each individual sample was placed in a 1.5 mL microcentrifuge tube and submerged in 99.5% acetone (PanReac AppliChem ITW Reagents, Castellar del Vallès, Spain) for 12 h to remove secondary metabolites that could interfere with PCR amplification [67]. After removing the solvent, the lichen thalli were left to air-dry completely. Samples were then mechanically disrupted using sterile glass pestles, following immersion of the microtubes in liquid nitrogen. DNA was extracted from the homogenized material using the Speedtools Tissue DNA Extraction Kit (Biotools, Madrid, Spain) according to the manufacturer’s instructions. As part of the extraction protocol, a pre-lysis incubation was performed at 56 °C for 3 h, with vortexing every 15 min to ensure sufficient tissue breakdown and thereby maximizing nuclear DNA release.

The fungi-specific primers ITS1-LM [68] and ITS2-KL [69] were employed to amplify the nuclear ribosomal internal transcribed spacer (nrITS) region. The nrITS region was selected because it has been proposed as the universal DNA barcoding marker for fungi [70], remains the most widely used marker in taxonomic and ecological mycological research at the genus level [71], and has proven effective as a first diagnostic approximation for discriminating and identifying lichen species in certain lineages in floristic surveys [72,73]. It includes the two variable spacers ITS1 and ITS2 and the intercalary, highly conserved 5.8S region. Its length typically ranges from 0.45 to 0.80 kb [74]. In the Ascomycota phylum, the weighted average of intraspecific ITS variability remains below the 3% threshold (1.96% ± 3.73, [75]). Refer to Appendix B for full details on the PCR protocol, purification, and sequencing.

##### Sequence Alignment and Phylogenetic Analysis

The newly generated sequences were manually edited in Geneious Prime 2025.0.3 (https://www.geneious.com), adopting the procedure detailed in Paul et al. [76] with minor adjustments (Appendix C). Chimera-check was done following the tree topology analysis [77].

To identify closely related taxa, each sequence was initially subjected to a BLAST+ v2.15.0 [78] similarity search against the UNITE database [79]. UNITE was selected as it is a curated, web-based reference database developed for the molecular identification of fungi, with a primary focus on the nrITS region [80]. For each sequence, the top ten hits were retrieved along with their associated Species Hypothesis (SH) codes, a system used by UNITE to guide species circumscription by clustering sequences into provisional species-level groups based on sequence dissimilarity thresholds of 0.5%, 1.0%, and 1.5%.

To perform the phylogenetic inference, reference sequences were retrieved from GenBank, ensuring appropriate representation of lichen taxa closely related to our samples, as well as species reported from Antarctica in general, and the Hurd Peninsula specifically, using the checklists of Øvstedal & Lewis Smith [8] and Søchting et al. [44] as references. This dataset was further supplemented with homologous sequences corresponding to the top BLAST results from the UNITE database (Table S1). For each sequenced specimen we included either its top hit based on similarity score values or one representative from the same species-hypothesis cluster at the 0.5% distance threshold, provided that a minimum coverage of 80% was achieved [81].

The newly generated sequences were subsequently aligned—alongside the reference sequences—with the MAFFT v7.490 [82,83] plugin available on Geneious Prime 2025.0.3 using default settings. The scoring matrix was set to 2000 PAM/k = 2, with a gap opening penalty of 1.53 and an offset value of 0.123. Ambiguously aligned regions were filtered out manually. Where portions of the nrITS region were missing, terminal gaps were padded with Ns to maintain alignment length.

Phylogenetic tree inference was done through maximum likelihood (ML) analyses in IQ-TREE 2 (version 2.4.0) [84]. Branch support was assessed with 1000 replicates each of ultrafast bootstrap approximation (UFBoot) [85,86] and the Shimodaira-Hasegawa-like approximate likelihood ratio test (SH-aLRT) [87]. The analyses were run under default settings and a minimum correlation coefficient of 0.99 for ultrafast bootstrap convergence. The best-fit substitution model was identified using the ModelFinder algorithm [88] implemented in IQ-TREE 2, based on the Bayesian Information Criterion (BIC). Bootstrap consensus trees were visualized with the program iTOL (Interactive Tree of Life) [89]. Each branch of the phylogenetic tree was accompanied by SH-aLRT and UFBoot support values. Branches with SH-aLRT support values ≥ 80% and UFBoot support values ≥ 95% [85] were regarded as well supported, following recommendations provided in the IQ-TREE manual for single gene trees [90]. The ascomycete species *Leotia lubrica* (Scop.) Pers. and *Neodermea acerina* (Peck) W.J. Li & K.D. Hyde was used as an outgroup [91].

#### 2.2.3. Integrative Taxonomy

Following the principles of iterative taxonomy proposed by Yeates et al. [92] and Leavitt et al. [93], morphological characters of the 102 specimens that underwent molecular analysis were re-evaluated in light of the phylogenetic results, guaranteeing both sources of data to be interpreted in a complementary and reciprocal manner [73,94]. The combination of molecular, morphoanatomical, and chemical data has proven to be an effective strategy for refining the taxonomic circumscription of morphologically ambiguous Antarctic lichens, either at a floristic survey covering multiple groups [81,95,96,97,98] or within a set of more closely related species [99,100,101,102,103].

Specimens whose sequences matched reference sequences in the phylogeny and whose morphological traits corresponded to the associated reference materials were confidently identified at the species level, whereas those whose sequences formed well-supported clades adjacent to known species and whose morphology matched the diagnostic traits of those species were likewise assigned accordingly. However, when morphological traits differed to varying degrees from those typically portrayed by the closest molecular match, the sample was designated as *affinis*, to indicate close affinity but not definitive identification. Samples whose sequences clustered with references identified only to the genus level, and for which phenotypic traits did not allow further resolution, were retained to that rank. These conservative approaches were adopted to avoid misidentification through overinterpretation of the data and to minimize the risk of unsupported calls when evidence was inconclusive, which could otherwise obscure databases and impede future taxonomic revisions [66,94].

### 2.3. Patterns in Diversity, Biogeographical Origin, Morphotype, and Reproductive Strategies

To further characterize the taxa identified from the four surveyed nunataks, we examined their taxonomic ranks, biogeographical distribution, morphotypes, and reproductive strategies to identify potential underlying patterns.

The total number of taxa identified was summed up according to their respective genus and family and subsequently visualized using a circular bar plot with the *ggplot2* R package [61].

Biogeographic categories were assigned based on four distributional groups (endemic, austral, bipolar, and cosmopolitan) following Sancho et al. [104]. The works of Fryday et al. [105], Garrido-Benavent and Pérez-Ortega [106], Olech [64], Øvstedal and Lewis Smith [8], Singh et al. [49], and Søchting et al. [44] were consulted for data on taxa distribution. Taxa classified as endemics encompass both strict Antarctic endemics and species found in the sub-Antarctic islands and/or the southernmost parts of South America, namely Patagonia and the Falkland Islands. The austral category refers to taxa restricted to the Southern Hemisphere but outside of tropical and subtropical areas. Bipolar taxa are those present in both boreal and austral regions and absent from tropical lowlands, at times also appearing on mountain systems distributed across both hemispheres, including non-polar zones such as mountainous temperate areas. Cosmopolitan taxa are widely distributed, occurring in more than one vegetation zone. In cases where taxonomic resolution did not extend beyond the genus level, no biogeographical assignment was made, and the distribution was marked as “undetermined”.

The morphotype categories considered in the analysis correspond to the three main traditional growth forms: crustose (including squamulose thalli of *Psoroma* and leprose thalli of *Lepra*, *Lepraria*, and *Pertusaria*), foliose, and fruticose.

Reproductive strategies were classified into three categories: sexual, asexual (rather referring to vegetative reproduction), or mixed. Taxa were considered sexual when reproduction of the mycobiont occurred through ascospores generated by apothecia or perithecia, asexual when only soredia or isidia were present, and mixed when evidence of both strategies was observed. Taxa lacking the aforementioned structures were listed as asexual, as they may reproduce through alternative modes of propagation, including the production of vegetative propagules (e.g., multicellular thalloconidia in *Umbilicaria africana* and *U. cristata*) or thallus fragmentation (e.g., *Pseudephebe minuscula*) [99,107].

Biogeographic, morphological, and reproductive data were visualized in a tree map using the *Treemapify* R package [108]. To ensure reliability in the biogeographical origin analyses, taxa designated as *affinis* or identified to family or genus level were excluded [43,44,109]. In contrast, the morphotype and reproductive plots included all taxa, regardless of taxonomic resolution.

### 2.4. Characterization of Lichen Flora on Antarctic Nunataks

#### 2.4.1. Lichen Checklist Compilation

Reports of lichen occurrence on Antarctic nunatak environments were gathered from various sources, primarily floristic surveys published as research papers or books. A thorough examination encompassing 31 literature resources was undertaken, spanning the period from 1966 to 2024: Filson [19], Øvstedal [110,111], Engelskjøn [112], Kappen [18], Øvstedal [113], Hale [114], Ryan et al. [41], Thor [115], Convey et al. [116], Pandey and Upreti [117], Øvstedal and Lewis Smith [8], Castello [109], Olech [64], Søchting et al. [44], Cannone [118], Convey and McInnes [119], Guglielmin et al. [120], Nayaka and Upreti [121], Green et al. [122], Øvstedal and Schaefer [123], Colesie et al. [124], Ertz et al. [125], Engelen et al. [126], Wagner et al. [127,128], Ochyra et al. [63], Pérez-Ortega et al. [129]. The BAS Antarctic Plant Database [130] was consulted to supplement the list of records provided in Convey and McInnes [119]. The selected time window still allowed us to ensure broad coverage across the main biogeographic regions and adequate taxonomic reliability, given the progressive improvement in floristic knowledge of the Antarctic continent. Only published surveys involving at least one lichen specialist in specimen collection and/or identification were considered to favor taxonomic accuracy. Studies in which taxonomic claims were based on photographic evidence rather than on the examination of physical specimens were excluded. Following the framework by Peat et al. [5], checklists written by C.W. Dodge that did not undergo a subsequent redetermination were disregarded. The collections conducted in Mount Durham, Scudder Mountain, and Thiel Mountains and surrounding areas in the Transantarctic Mountains, as detailed in Dodge [131], Dodge & Baker [132], and Dodge & Rudolph [133], and those that were later reviewed in Øvstedal & Lewis Smith [8], were included. Additional occurrence records were obtained from genus-specific monographs.

The concept of nunatak adopted was ecological and biogeographical rather than geomorphological, with isolation from surrounding ice-free exposure—to varying degrees—by permanent glacial ice being the defining characteristic. Consequently, surveys of individual nunataks consisting of mountain peaks or knobs encircled by permanent ice [18,44,116,123,126] as well as those conducted on groups of nunataks within mountain ranges or massifs [19,20,41,63,109,110,111,112,113,115,117,118,119,120,121,122,124,125,127,128,129], all differing in height and in the extent of exposed surface above the surrounding glacial ice, were treated as proxies for nunatak environments (*sensu* Convey et al. [15]). For the flora of King George Island [64], the author’s categorization of nunatak was respected. It includes both altitudinal (rock needles and ridges with small summit areas and steep sides) and topographical types (peaks with broad, flat summits), following the descriptions made previously by Ochyra [134]. A complete list of the nunatak sites considered in this study is provided in Table S4.

**Figure 2:**
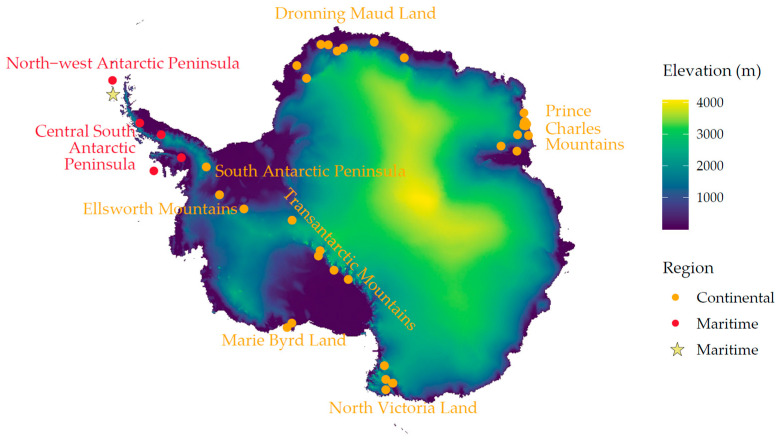
Map of Antarctica highlighting all nunataks considered for the present study. Each nunatak is represented with a colored dot: orange indicates nunataks located in the continental region, whereas red denotes those situated in the maritime region. The yellow star marks the position of the newly sampled nunataks in the Hurd Peninsula (maritime region). The color gradient illustrates elevation, ranging from low-elevation permanent ice shelves (dark blue) to the highest mountainous areas (yellow). The Antarctic Conservation Biogeographic Region associated with each nunatak is shown by the labels. For more details, see Table S4.

Geographical coordinates for each individual nunatak—or groups of nunataks—included in the checklist were obtained from the original publications whenever provided. When only the sampling place name was given, the corresponding coordinates were retrieved from the SCAR Composite Gazetteer of Antarctica (https://data.aad.gov.au/aadc/gaz/scar/, accessed on 10 December 2025) or the Norwegian Polar Institute’s placement database (https://data.npolar.no/placename, accessed on 10 December 2025). If the points appeared on ice- or snow-covered surfaces when visualized in Google Earth, they were repositioned to match the exposed collection areas described in the original sources. When no coordinates were available from any of these repositories, localities were manually georeferenced using published maps and written descriptions. All gathered points were then geographically positioned on the surface elevation map from the Bedmap3 project [135] (Figure 2). Coordinates were first reprojected to EPSG:3031 and then plotted using the *terra* [60], *tidyterra* [136], and *ggplot2* [61] R packages.

The checklist was complemented with lichen records from deglaciated environments other than nunataks retrieved from existing databases on Antarctic biodiversity as well as from the published literature on Antarctic lichen flora, as explained in Appendix D. The taxonomic diversity of the entire dataset was visualized at the genus level using the *WordCloud* R package [137]. Furthermore, a bar plot was created with the *ggplot2* R package [61] to highlight the taxa that are not confidently identified to species level in the nunatak flora (Figure S3).

Biogeographical and morphological assignments for each taxon were verified. When these data were not provided in the source, categorization was inferred following the procedure detailed in Section 2.3. The crustose growth form was made to house nanofruticose specimens as well as the inconspicuous thalli of parasitic lichenized fungi, on top of the additional morphotypes listed in Section 2.3, while the fruticose also encompassed filamentous (e.g., in *Rhacodiopsis rupestris* and *Zahlbrucknerella* spp.) and composite thalli of *Cladonia* specimens. Two bar plots were generated to visualize the biogeographical and morphological assignments, as previously outlined.

#### 2.4.2. Statistical Analyses

Contingency analyses were conducted to determine whether significant differences in biogeographic origin and morphotype occurred between nunatak and non-nunatak areas overall, and to evaluate variation in relative proportions at regional and local scales. Chi-squared tests of independence were applied when assumptions were met, whereas Fisher’s exact test was used when expected cell counts were low. Statistical significance was assessed at *p* < 0.05 for all analyses.

#### 2.4.3. Haplotype Network Analysis

To gain preliminary insights into the genetic structure of lichen species occurring in both nunatak and non-nunatak environments across Antarctica, we conducted an initial exploratory analysis of haplotype distributions using the subset of shared species identified in the compiled checklist (see Section 2.4.1).

ITS sequence data were available in GenBank for 116 of the 176 candidate species. Among these, 86 species included at least one sequence derived from Antarctic samples. For these 86 taxa, the geographic provenance of each Antarctic sequence was verified by consulting the original publications in order to determine, whenever possible, whether samples originated from nunatak or non-nunatak environments, either from maritime or continental Antarctica. To balance data reliability with the need to ensure a minimum level of taxonomic coverage, only species represented by sequences from at least two habitat-region categories (non-nunatak in maritime Antarctica, nunatak in maritime Antarctica, non-nunatak in continental Antarctica, and nunatak in continental Antarctica) were retained for downstream analyses, provided that each group included at least two sequences. To further minimize potential bias arising from uneven sampling, we quantified the imbalance among habitat-region categories for each species as the ratio between the largest and smallest sample sizes (n_max_/n_min_) [138]. Only species for which represented groups were well balanced (imbalance ratio ≤ 2) or moderately balanced (2 < imbalance ratio ≤ 5) were evaluated. This resulted in a final dataset comprising eight species (Table S5): *Acarospora gwynii*, *Austrolecia antarctica*, *Lecanora fuscobrunnea*, *L*. *physciella*, *Lecidea cancriformis*, *Lecidella siplei*, *Pseudephebe minuscula*, and *Usnea lambii*.

Sequences were aligned using MAFFT v7.490 [82,83] implemented in Geneious Prime 2025.0.3, with default parameters (see Section 2.2.2). All sequences were trimmed to the longest common overlapping region. Ambiguously aligned regions were removed manually, as were isolated positions containing gaps or ambiguous base calls. Statistical parsimony networks were constructed using a 95% parsimony probability criterion [139], as implemented in the R package *pegas* [140].

## 3. Results

### 3.1. Lichen Identification in Hurd Peninsula Nunataks

Overall, the study of the collected specimens recovered one lichenicolous fungus and 71 lichenized fungi, of which 39 were confidently identified to species level (Table 1). Several records remain tentative: 11 were assigned only to genus, two to family, 17 were treated as *affinis*, and two were recognized as morphotypes. Should future work confirm that the uncertain *affinis* entities match their current identifications, the documented species richness could rise to 49. As it stands, floristic richness remains lower than that reported for the Hurd Peninsula, which, upon revision, harbors 197 total taxa and 184 confidently identified species [44].

#### 3.1.1. Phenotypical Characterization of Lichen Samples

*Umbilicaria cristata* is reported for the first time from Livingston Island. Thalli were collected on rock crevices of a north-facing wall in Nunatak del Castillo. A clarification is required regarding the specimen identified as *Thelenella* aff. *mawsonii*, which could alternatively represent an atypically thin thallus of *T. antarctica*. The thallus was thin, somewhat effuse and rimose, dark grey, and verrucose, with scattered white to pale grey, warty areolae, differing from the typically pale greenish-grey to brown-green thallus described for *T. mawsonii*. Perithecia were mostly solitary, rarely occurring in pairs within the same areola but developing on distinct lobules. They showed a black involucrellum restricted to the uppermost part, and in more developed ascomata the ostiole was excavate. Perithecia slightly protruded through the surrounding hypothallus, whereas in *T. antarctica* they are usually immersed within the areolae [8,64]. Nevertheless, their degree of emergence did not reach the almost superficial condition reported for *T. mawsonii* [8,64,141]. Examination of ascus spore content did not resolve species identity, as spore numbers corresponded to published descriptions of both taxa. Ascospores were muriform, hyaline, and displayed a number of transverse and longitudinal septa that fell within the ranges reported for both *T. antarctica* and *T. mawsonii*. Although mature spores of *T. antarctica* are reported to be broader and longer than those of *T. mawsonii*, spore size was considered of limited diagnostic value here, as the observed spores may not have been fully developed. Given the scarcity of confirmed records of *T. mawsonii* in the South Shetland Islands—restricted to a few observations from King George Island [64]—and the comparatively broader distribution and higher abundance of *T. antarctica*, including previous reports from the South Bay area [43,44], identification as *T. antarctica* remains plausible. However, the combination of thallus structure, together with the moderately emergent perithecia and sunken ostiole, supported the provisional assignment of this specimen to *Thelenella* aff. *mawsonii*.

#### 3.1.2. Molecular Identification

Of the 102 specimens selected for molecular analysis, 63 yielded usable sequences: 52 bidirectional and 11 unidirectional. The remaining 39 exhibited poor-quality electropherograms and their taxonomic classification relied solely on the initial morphological examination.

Among the 63 samples that were successfully sequenced, 62 matched lichenized fungal taxa according to BLAST results (Table S1) and were therefore retained for phylogenetic analysis. The remaining sequence corresponded to a non-lichenized endophytic fungus, showing >99% similarity to 11 clone sequences deposited in GenBank. These clones belong to an uncultured member of the order *Pertusariales* and were isolated from the moss *Chorisodontium aciphyllum*, collected on Ardley Island (South Shetland Islands) [142]. In our study, this fungus was obtained from a specimen morphologically identified as *Lepra excludens* collected at Nunatak del Castillo. Given its non-lichenized nature and the lack of closely related lichenized taxa, the sequence was excluded from downstream phylogenetic reconstruction. Nevertheless, its identification was considered reliable based on the BLAST searches against the UNITE database, which returned nine top hits with 100% sequence identity and 100% coverage. Although the fungus was detected within lichen thallus tissue (i.e., endolichenic), we retained the designation used in the original publication (uncultured endophytic fungus) to maintain nomenclatural consistency pending formal taxonomic assignment.

**Figure 3:**
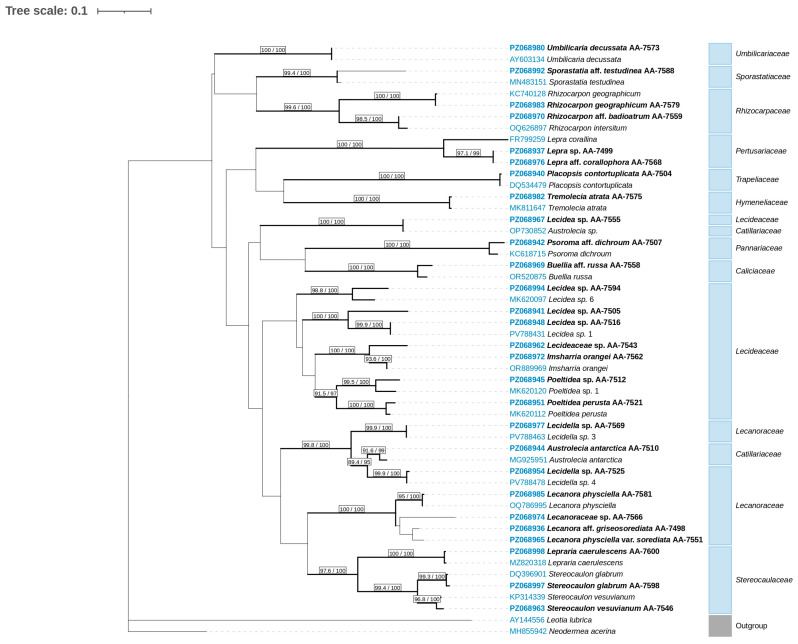
Simplified phylogenetic tree depicting phylogenetic relationships among lichenized fungi retrieved in the survey and closest related taxa, inferred using maximum likelihood based on the nrITS marker. Branch support values are shown above the corresponding branches and are given as SH-aLRT/UFBoot. Only branches supported by both SH-aLRT ≥ 80% and UFBoot ≥ 95% display support values and are depicted in bold. Branch tips are labelled with GenBank accession numbers (blue) and taxon names (black). Newly generated sequences are printed in bold. Family affiliations are indicated to the right of the tree tips. *Leotia lubrica* and *Neodermea acerina* were used as outgroups. The scale bar represents 0.1 substitutions per site. For a detailed version of the phylogenetic tree, see Figure S2.

The phylogenetic analysis was based on a data matrix comprising 353 nrITS sequences, including 62 newly generated sequences, 289 reference sequences, and two outgroups (Table S2). The final alignment spanned 540 bp. According to the BIC, the best-fit substitution model was TIM2+F+R6. Based on the resulting phylogeny, a simplified dataset comprising 52 sequences was constructed for clarity (Figure 3), including 28 newly generated sequences, 22 reference sequences, and two outgroups. Newly generated sequences were selected to ensure that each taxon retrieved in this study was represented. When more than one sequence per taxon was available, a single representative was retained. In cases where sequences identified only to the genus level were recovered in different clades, one representative from each clade was selected. For each selected original sequence, a single reference sequence was included, corresponding to the closest match in the detailed phylogenetic tree. The alignment of this reduced dataset spanned 527 bp. The best-fit substitution model according to the BIC was TN+F+I+G4. Both ML phylogenetic trees—the one based on the complete dataset (Figure S2) and that derived from the simplified dataset (Figure 3)—revealed strong coherence at the family and genus level. Most families housing the newly generated sequences were well supported. However, sequences of the present study assigned to *Lecanoraceae* and *Lecideaceae* were recovered in polyphyletic positions. Similarly, most genera encompassing the newly generated sequences were recovered as well-supported, monophyletic clades (Figure S2). Nevertheless, several genera displayed paraphyletic placements, including *Austrolecia*, *Buellia*, *Lecanora* s. lat., *Lecidea*, and *Lecidella*. The genus *Austrolecia* (family *Catillariaceae*), which currently includes a single described species, was recovered in three distinct positions within the order *Lecanorales*. This pattern reflects ongoing uncertainty in its phylogenetic placement.

Across the phylogeny, the newly generated sequences were distributed among sixteen genera (Figure S2). Twenty of them were recovered within thirteen well-supported clades together with reference sequences confidently identified at the species level: *Austrolecia antarctica*, *Buellia russa*, *Imsharria orangei*, *Lecanora physciella*, *Lepraria caerulescens*, *Placopsis contortuplicata*, *Poeltidea perusta*, *Psoroma dichroum*, *Rhizocarpon intersitum*, *R. geographicum*, *Stereocaulon glabrum*, *Tremolecia atrata*, and *Umbilicaria decussata*. An additional eight were affiliated with reference sequences assigned to genus level: *Austrolecia* sp., *Lecidea* sp., *Lecidella* sp., *Poeltidea* sp. and *Stereocaulon* sp. The remaining 34 sequences occurred either as singletons or grouped amongst them outside clades containing identified reference material. Of these, 22 showed the closest affinity to a reference sequence assigned to a described species and were therefore provisionally designated with the qualifier aff. pending morphological confirmation. The other 12 sequences were most closely related to reference sequences identified only at the genus level and were provisionally assigned to the corresponding genus accordingly.

#### 3.1.3. Taxonomic Delimitation Based on Integrative Evidence

The 62 provisional identifications obtained from the phylogenetic study were cross-checked for congruence with diagnostic phenotypic features (Table 1). Overall, the integrative approach enabled the identification of 21 specimens at the species level. Sixteen of these matched eight species previously determined through phenotypical analysis (*Austrolecia antarctica*, *Lecanora physciella*, *Placopsis contortuplicata*, *Poeltidea perusta*, *Sporastatia testudinea*, *Stereocaulon glabrum*, *Tremolecia atrata*, and *Umbilicaria decussata*), while five revealed taxa that had not been recognized based on morphology alone: *Imsharria orangei*, *Lecanora physciella* var. *sorediata*, *Lepraria caerulescens*, and *Stereocaulon vesuvianum*. An additional eight sequences were designated as species *affinis*, four of which represented three previously unrecognized taxa through morphological examination alone: *Buellia* aff. *russa*, *Lepra* aff. *corallophora*, and *Psoroma* aff. *dichroum*. Twenty-two sequences were identified to genus level and 11 to family level.

### 3.2. Lichen Taxonomic Diversity and Traits in Hurd Peninsula Nunataks

**Figure 4:**
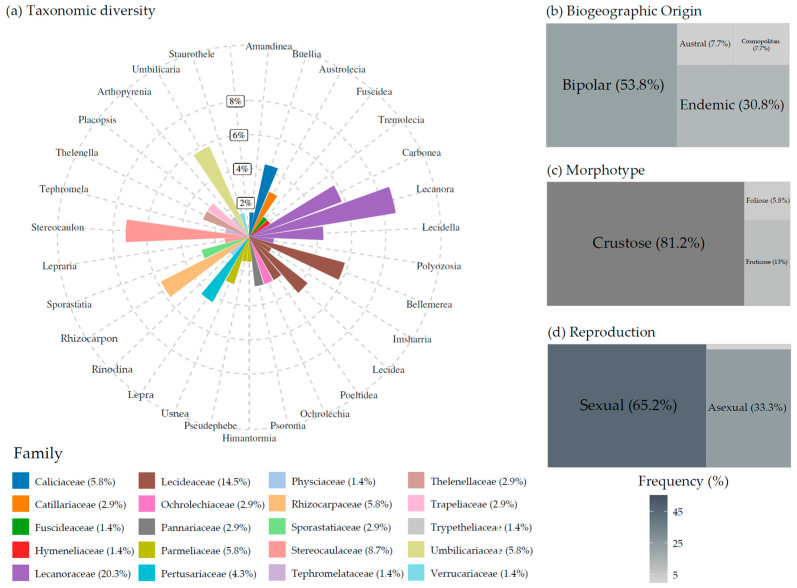
Summary of the characteristics of the nunataks sampled in this study: (**a**) Circular bar plot illustrating the number of species (represented by bar height) per genus (labels on the circular plot). The color of each bar indicates the family to which each genus belongs. (**b**) Tree map displaying the biogeographic origin of the confidently identified species and varieties. (**c**) Tree map depicting the morphotype of all identified taxa. (**d**) Tree map showing the reproductive strategy of all taxa. The lightest grey section corresponds to Sexual and Asexual (1.5%).

Across the four surveyed localities, the lichenized fungal samples encompassed 30 genera, 20 families, and 11 orders (Figure 4a, Table 1 and Table S3). Overall, the lichen flora was dominated by the order *Lecanorales*, including 40.6% of the identified taxa (46.2% when accounting for the confidently identified species alone). The families *Lecanoraceae* and *Lecideaceae* exhibited the greatest generic diversity, each comprising four genera and seven and five species, respectively (Figure 4a, Table S3). These were followed by *Stereocaulaceae*—housing two genera and five species—and *Umbilicariaceae*—with one genus and four species. Several families were minimally represented in the overall dataset: *Fuscideaceae*, *Hymeneliaceae*, *Physciaceae*, *Tephromelataceae*, *Trypetheliaceae*, and *Verrucariaceae* were each represented by a single taxon.

The biogeographic profiles (Figure 4b) of the confidently identified species recorded revealed that 53.8% had a bipolar distribution, making it the most represented category across the surveyed sites. In contrast, cosmopolitan and austral taxa accounted for 7.7% of the total diversity each. Endemics comprised 30.8% of the total set of species.

The crustose morphotype was by far the most dominant, accounting for 81.2% of all recorded taxa (Figure 4c). This was followed in abundance by fruticose (13.0%) and, to a lesser extent, foliose growth forms (5.8%). Fruticose lichens were primarily represented by *Himantormia lugubris*, several species belonging to the genus *Stereocaulon*, *Usnea aurantiacoatra*, and *U. antarctica*. All foliose specimens collected belonged to the genus *Umbilicaria*.

A total of 65.2% of taxa reproduced exclusively through sexual means, while 33.3% did so solely via asexual (vegetative) strategies (Figure 4d). Of the total taxa, 22.5% developed soralia. Only one specimen, identified as *Buellia* aff. *soredians*, exhibited reproductive flexibility, being capable of reproducing both sexually and asexually.

### 3.3. Lichen Diversity in Antarctic Nunatak and Non-Nunatak Environments

#### 3.3.1. Taxonomic Diversity of Lichen Assemblages

**Figure 5:**
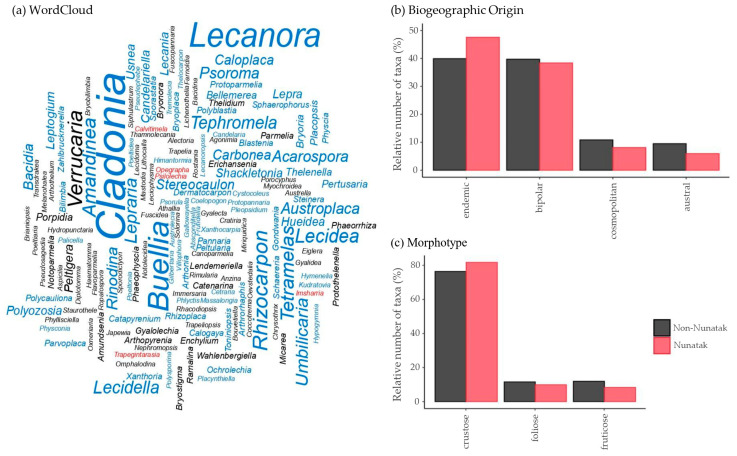
Characteristics of the Antarctic lichen flora occurring on nunatak and non-nunatak environments: (**a**) Word cloud showing the genera occurring exclusively on nunataks (red), exclusively in non-nunatak areas (black), or in both environments (blue). Label size reflects the number of taxa within each genus. (**b**) Bar plot depicting the biogeographic origin of species, subspecies, and varieties in nunatak (red) and non-nunatak areas (black). (**c**) Bar plot illustrating the morphotypes of all reported taxa in nunatak (red) and non-nunatak areas (black).

As for the overall Antarctic lichen flora, 73.3% of the taxa recorded from nunataks (187 out of 255 total taxa) were confidently identified at the species, subspecies level, or variety compared with 91.1% for non-nunatak areas (509 out of 559 total taxa) (Figure S3). The number of taxa showcasing uncertain classifications—such as *affinis* and *confer*—or those that remain identified only to genus, family, or class level was notably higher in nunatak areas (Figure S3). In the Antarctic flora overall, the most species-rich genus was *Cladonia* (35 species), followed by *Lecanora* (24 species, one subspecies, and one variety) and *Buellia* (23 species) (Figure 5a). On nunataks in particular, the most speciose genera were *Lecanora* (13 species and one variety), *Buellia* (9 species), and *Rhizocarpon* (8 species).

Cases of confidently identified species that have so far been recorded exclusively from nunataks amounted to 11: *Amandinea clearyi* (recorded from the Alexandra and Rockefeller Mountains, Marie Byrd Land), *Calvitimela uniseptata* (found in Vestfjella, Dronning Maud Land), *Carbonea antarctica* (collected from the Rockefeller Mountains), *Carbonea vitellinaria* (recorded from the Mount Kyffin area, Transantarctic Mountains), *Imsharria orangei* (reported in the present study for the nunataks of Hurd Peninsula), *Lecanora pseudephebes* (first recorded in the Mount Jackson area, south-eastern Antarctic Peninsula, and later observed in the Alexandra and Rockefeller Mountains), *Opegrapha edsonii* (described from Edson Hill in the Heritage Range, Ellsworth Mountains), *Psilolechia lucida* (found in the Marion nunataks, Charcot Island), *Schaereria albomarginata* (discovered in Eternity Range, Palmer Land), *Tetramelas filsonii* (described from the North and South Masson Range, Mac. Robertson Land), and *Trapegintarasia antarctica* (reported from the Sør Rondane Mountains, Dronning Maud Land). Five of these species belonged to genera restricted to nunatak environments and represented by a single species in our dataset (namely *Calvitimela*, *Imsharria*, *Opegrapha*, *Psilolechia*, and *Trapegintarasia*; see Figure 5a). In contrast, 77 genera were found exclusively in non-nunatak areas. The remaining 87 genera were shared between nunatak and non-nunatak areas, indicating that most lichens occurring on Antarctic nunataks belong to a broader pool of taxa widely present in non-nunatak environments. Some of the more widespread species among nunataks from this shared pool include *Acarospora gwynnii*, *Buellia frigida*, *Candelariella flava*, *Carbonea vorticosa*, *Lecidea cancriformis*, *Physcia caesia*, *Pleopsidium chlorophanum*, *Pseudephebe minuscula*, *Rhizoplaca melanophthalma*, *Umbilicaria decussata*, *Usnea lambii*, and *Xanthoria elegans*.

Comparison of species composition between nunatak and non-nunatak floras across regions revealed clear trends. In continental Antarctica, 68.3% of the confidently identified species recorded on nunataks were found in non-nunatak areas as well (99 out of 145 species). Contrastingly, in the maritime region, this proportion was much lower, reaching no more than 26.5% (125 out of 471 species). Moreover, while 51.0% of the species recorded outside nunataks in continental Antarctica occurred on nunataks too (74 out of 145 species), the overlap dropped to only 25.5% in maritime Antarctica (120 out of 471 species). This pattern was also evident at a local scale in Hurd Peninsula, where 20.1% of the confidently identified species from non-nunatak sites were found on the nunataks in the area, including those surveyed in this study, as well as Moores Peak (37 species out of 184). This proportion would increase to 22.8% if the *affinis* taxa from the nunataks were to be confirmed as distinct species.

Out of the 24 orders reported overall, considering only confidently identified species, subspecies, and varieties, five were absent from nunataks: *Chaetothyriales* (represented by *Rhacodiopsis rupestris*), *Graphidales* (containing only *Gyalidea mayaguezensis*), *Gyalectales* (represented by *Gyalecta pezizoides*), *Lichenotheliales* (*Lichenothelia antarctica*), and *Trypetheliales* (including *Arthopyrenia maritima* and *A. praetermissa*). Fifty-five families were recorded across nunatak and non-nunatak areas, of which one third (16) have not been found on the former. Conversely, two families have thus far been reported exclusively from nunatak settings: *Opegraphaceae* (represented by *Opegrapha edsonii*) and *Psilolechiaceae* (containing *Psilolechia lucida*).

#### 3.3.2. Biogeographical and Morphological Patterns

Contingency analyses revealed no significant differences in overall biogeographic composition between nunatak and non-nunatak environments across Antarctic regions (*Χ*^2^ = 4.979, df = 3, *p* = 0.173) (Figure 5b). Likewise, no significant differences were detected between maritime and continental nunataks when analyzed separately by region (*Χ*^2^ = 3.742, df = 3, *p* = 0.291), although the endemic distribution showed a higher proportion in nunataks (Figure S4).

In the maritime Antarctic region, endemic and bipolar taxa occurred in comparable proportions both on nunataks (41.5% and 43.9%, respectively) and in non-nunatak environments (38.4% and 40.7%, respectively). Consistently, contingency analyses indicated no significant differences in biogeographic composition between sites within the maritime region (*Χ*^2^ = 2.485, df = 3, *p* = 0.478). In contrast, in continental Antarctica endemic taxa constituted the largest share of the nunatak flora (53.1%), followed by bipolar taxa (33.3%) (Figure S4). A similar pattern was observed in continental non-nunatak environments, where endemic taxa also exceeded bipolar taxa in frequency (47.9% vs. 36.1%, respectively). As could be foreseen, no significant differences emerged between nunatak and non-nunatak habitats in continental Antarctica (*Χ*^2^ = 0.667, df = 3, *p* = 0.881). Because of small expected frequencies in the austral category, Fisher’s exact test was additionally performed, which confirmed the absence of significant differences (*p* = 0.901). Moreover, no statistically significant differences were identified between maritime and continental non-nunatak environments (*Χ*^2^ = 6.395, df = 3, *p* = 0.094).

At the local scale, no significant differences in biogeographic composition were found between the flora of the Hurd Peninsula lowlands and mountainous ice-free areas and that of its nunataks, including the four nunataks examined in the present work as well as Moores Peak (Fisher’s exact test, *p* = 0.381), despite the comparatively higher proportion of bipolar taxa observed on nunataks (52.4%) relative to that other ice-free areas of the peninsula (37.0%; Figure S5). Similarly, no significant differences were detected between Hurd Peninsula nunataks and maritime nunataks overall (*p* = 0.784), between Hurd Peninsula nunataks and continental nunataks (*p* = 0.106), or between Hurd Peninsula nunataks and maritime non-nunatak environments (*p* = 0.580).

Contingency analyses yielded no significant differences in overall morphotype composition between nunatak and non-nunatak environments across Antarctic regions (*Χ*^2^ = 3.190, df = 2, *p* = 0.203), despite the higher representation of the crustose morphotype on nunataks compared to non-nunatak areas (Figure 5c). Conversely, the fruticose morphotype appeared slightly less represented on nunataks.

#### 3.3.3. Haplotype Network Structure

Figure S6 depicts the statistical parsimony networks illustrating genetic relationships among haplotypes for the eight candidate species occurring in both nunatak and non-nunatak environments across Antarctica. On average, eight haplotypes per species were identified, ranging from four in *Acarospora gwynnii* and *Pseudephebe minuscula* to 22 in *Lecidea cancriformis*.

Overall, no clear or consistent segregation of haplotypes was observed between nunatak and non-nunatak sites within either maritime or continental regions, nor between maritime and continental samples irrespective of habitat. In the few cases where some degree of grouping was observed, either between nunatak and non-nunatak samples (e.g., *Acarospora gwynnii*) or between maritime and continental samples (e.g., *Lecidella siplei* and *Pseudephebe minuscula*), the limited number of sequences per haplotype prevents drawing robust conclusions.

Most haplotypes within each species differed by a single mutation. However, in *Lecanora physciella*, markedly higher levels of divergence were consistently observed (e.g., 26 mutations between haplotypes h5 and h6). In five of the eight species, at least one haplotype was shared between nunatak and non-nunatak samples. These shared haplotypes corresponded to the most frequent and widely distributed ones within each species.

Unique haplotypes restricted to nunatak samples were detected in all species, although they were generally represented by a few sequences. Haplotypes h5 to h8 of *L. physciella* correspond to sequences generated in this study, each representing a previously unrecorded haplotype.

## 4. Discussion

### 4.1. Nunataks of the Hurd Peninsula

The following section synthesizes several ecological features that were consistently observed in situ across the four nunataks surveyed on Hurd Peninsula. These direct field observations provide a robust framework for interpreting the patterns in taxonomic diversity and growth-form composition reported in this work and for contextualizing the environmental setting of the study area. Lichen communities recorded across the sampled sites were restricted to niches scattered throughout sheltered crevices, wind-swept crests and flat surfaces. They seemed to benefit from microtopographic features that provide shelter from the direct incidence of wind-blown ice-crystals, release thermal radiation, and ensure a frequent input of moisture through summer snowmelt, although naturally there are underlying interspecific differences in tolerance and resilience. In addition, the frequent cloud cover observed at the surveyed localities may provide a relatively constant moisture supply and limit excessive insolation, which in turn could enable lichens to colonize more open and exposed substrata [18]. The absence of vascular plants from all four surveyed nunataks is noteworthy, particularly in light of previous reports of *Colobanthus quitensis* and *Deschampsia antarctica* on the north-facing slope near the summit of the neighboring Reina Sofia Peak, where they occur in association with *Placopsis contortuplicata* and *Stereocaulon alpinum* [43]. Based on our direct field observations, this peak may represent the distributional limit for vascular plants in the area, not only in altitudinal terms, but also with respect to broader environmental constraints. Bryophyte cover, typically extensive in moister environments within maritime Antarctic ecosystems [1], was sparse in comparison to coastal areas. Only small and scattered moss cushions were observed occasionally, confined to sheltered rock crevices. Similarly, terricolous and fruticose lichens such as *Lepraria caerulescens*, *Ochrolechia frigida*, *Psoroma* spp., and *Stereocaulon* spp. were restricted to microsites where soil and moisture could accumulate. The overall lichen taxonomic richness recorded across the four nunataks was markedly lower than that observed in lower-altitude coastal areas of the Hurd Peninsula, consistent with previous observations from Moores Peak [44].

This reduced diversity further supports the notion that nunataks are subjected to more severe environmental conditions, acting as strong environmental filters. This interpretation is consistent with findings from Fernández-Martínez et al. [48], who reported that levels of total organic carbon and total nitrogen in nunatak soils and rocks of both Moores Peak and MacGregor Peak were markedly lower than those found in more developed coastal soils at Sally Rocks (Hurd Peninsula). Instead, these values more closely resembled those measured in recently deglaciated substrates at the terminus of Hurd Glacier, or even in soils of the Dry Valleys in South Victoria Land. These conditions suggest that lithobiontic and edaphic microbial activity, and consequently nutrient availability, must be strongly constrained in nunatak environments of the Hurd Peninsula, likely limiting the establishment and growth of cryptogamic macroflora.

Several taxonomic findings are noteworthy due to their novelty. Notably, *Umbilicaria cristata*—an Antarctic endemic species only rarely recorded in both continental and maritime Antarctica—is here reported for the first time from Livingston Island, specifically from Nunatak del Castillo. *Stereocaulon vesuvianum* is likewise newly recorded from the island, further expanding its known distribution in the maritime Antarctic region. Moreover, the occurrence of *Imsharria orangei* represents the first record of this recently described species for Antarctica. Previously, it had only been reported from the Falkland Islands, where it occurs at or near mountain summits [143]. This finding suggests that *I. orangei* may have a broader distribution in high-elevation environments on Livingston Island, and potentially elsewhere in maritime Antarctica.

The remarkable biodiversity of Nunatak del Castillo compared with the other three surveyed sites (Table 1) is likely linked to its relatively extensive exposed rock surface, consistent with the positive relationship between plant cover and nunatak size reported by Ryan and Watkins [42] and the species-area relationship [144,145]. Additionally, the area displays a highly heterogeneous microtopography, which further increases the diversity of available microhabitats through a mosaic of exposures, orientations, and slopes. Such environmental heterogeneity is expected to promote species coexistence by providing a wide range of ecological niches and reducing direct competition.

Differences in mineral composition and rock stability among the surveyed nunataks may further contribute to the observed patterns in taxonomic diversity. Nunatak del Castillo belongs to the Mount Bowles Formation of the South Shetland Islands magmatic arc [146,147] and is composed of relatively coherent volcanic rocks with massive and autoclastic textures [62]. In contrast, the other sites are dominated by sedimentary formations. Napier Peak consists of shales and interbedded fine sandstones, mudstones, and conglomerates [146,147], assigned to the Napier Peak Breccias or the Moores Peak Member lithostratigraphic units of the Miers Bluff Formation [148]. Cerro Mirador is part of the Moores Peak Member unit, which is characterized by massive sedimentary breccias with sandstone and mudstone clasts [148]. MacGregor Peak is a Johnsons Dock Member of the Miers Bluff Formation and is dominated by turbiditic deposits, with outcrops heavily frost-shattered into unstable shales [48]. Although all sites are subject to periglacial processes (i.e., fragmentation and weathering), the comparatively greater stability of the rock surfaces at Nunatak del Castillo may provide more persistent substrates, thereby facilitating the long-term establishment and persistence of lichen communities. Lithological differences are likely to influence water retention, which would also affect lichen colonization.

Potential differences in exposure history may also have contributed to the observed patterns. As the most prominent of the four surveyed nunataks, Nunatak del Castillo may have deglaciated earlier and remained ice-free for longer periods, thereby allowing more time for propagule arrival and long-term community development. For comparison, the highest exposed rocks of Napier Peak—barely protruding several tens of meters above the surrounding ice surface—have been estimated to have become ice-free after the Last Glacial Maximum, at ca. 14 ka [149]. Given its greater prominence (over 100 m higher), Nunatak del Castillo may therefore have experienced a longer period of exposure.

An interesting feature of Nunatak del Castillo was its summit: a flat surface exposed to strong winds that remained entirely free of snow during our surveys and supported a well-developed community of fruticose and muscicolous lichens with high overall cover. A similar pattern was noted by Hovenden and Seppelt [150] on a knoll on Clark Peninsula (Windmill Islands Oasis, Wilkes Land), where windswept upper surfaces were the first to become snow-free in summer, extending the period during which the substratum is exposed to incident radiation and bioavailable moisture. In contrast, downslope areas and sheltered rock crevices on the leeward faces of the outcrop accumulated deeper and longer-lasting snow, leading to delayed melting and an overall shorter exposure time. The situation at Nunatak del Castillo appears consistent with these observations: lower parts of the north-facing slopes and a northwest-oriented gully of Nunatak del Castillo retained banks of seasonal snow, likely due to the combined effect of local topography favoring snowdrift, wind-driven deposition, and reduced incident radiation. As a result, these areas may experience a shorter period of exposure compared with the summit and other windswept niches, potentially limiting growth and colonization opportunities.

What appeared to be shell debris was found at the summit of Nunatak del Castillo. We hypothesize that some rocks might occasionally be used as bird perches by cliff-breeding birds like petrels or South Polar skuas. Even then, the direct and regular influence of birds (namely an increased external input of ammonia) can still be considered close to non-existent, as no colonies appear to be nearby. Such is the case for the other three explored sites. This is further evidenced by the absence of ornithocoprophilous lichen specimens typically found on or adjacent to intensely eutrophicated substrata. Nitrophobous fruticose macrolichens, however, flourish plentifully. No nanofruticose growth forms were encountered, which aligns with previous observations that such morphologies tend to occur primarily in comparatively eutrophicated environments [151,152]. Oligotrophy is not necessarily extreme, however, and atmospheric ammonia deposition could well occur. NH3 may be volatilized from biogenic sources, most notably the small penguin rookery along the coast of Caleta Argentina and the much larger colony at Hannah Point, located approximately 14 km away. Additional contributions may also originate from inland flying-bird colonies at nearby sites, such as Reina Sofia Peak. Once emitted, NH3 can be deposited with varying frequency and intensity depending on prevailing wind patterns [28] and may be transported over considerable distances [153,154,155,156].

As noted above, frost shattering was common across all four nunataks and likely disrupts vegetation through repeated freeze–thaw cycles. The substrate instability inherent in this periglacial phenomenon can continually disturb established flora and hinder further colonization [6,23,157]. Nonetheless, frequent rock breakage may in turn favor recolonization, as fallen, lichen-covered fragments can act as propagule sources for lower slopes, thereby helping maintain local floristic composition.

The predominance of sexually reproducing taxa in our material aligns with patterns frequently reported from extreme environments [158]—notably at the earlier stages of succession of lichens colonizing recently deglaciated moraines in South Bay [157,159]—where long-distance dispersal of fungal spores and the incrementation of genetic diversity may facilitate the establishment of pioneer populations on newly exposed substrates. Although metabolically costly, reliance on sexual reproduction enables lichens to endure adverse conditions and maintain genetic variability. It is noteworthy, however, that sorediate specimens, despite accounting for 22.5% of the taxa reported, were widespread across the study area and stood out in terms of biomass. Relatively large thalli of sorediate crustose lichens, compared to co-occurring crustose taxa, were commonly found covering extensive areas of the collected rock samples. Vegetative propagules such as soredia and isidia enable the simultaneous dispersal of both symbionts and promote the rapid establishment of new thalli. Owing to their greater size and mass relative to fungal ascospores, they are better suited for short-distance dispersal. On nunataks, such propagules may be particularly advantageous for effectively colonizing newly ice-free surfaces at the local level as gelifraction and glacial retreat progress.

The unresolved status of several specimens—whether treated as *affinis*, assigned to higher taxonomic ranks, or recognized only as morphotypes—reveals considerable taxonomic potential still hidden within these nunataks, indicating that additional, yet undescribed diversity remains to be uncovered.

### 4.2. Lichen Flora of Antarctic Nunataks

Most lichens found on Antarctic nunataks belong to a set of common taxa shared with non-nunatak environments. The floristic differences between the two settings primarily reflect a decline of the species typical of coastal and low-elevation assemblages, rather than the emergence of new taxonomic units with increasing isolation, mirroring the pattern described along the Antarctic Peninsula with rising latitude [5]. It remains uncertain whether the few species recorded exclusively from nunataks are truly restricted to these environments—particularly the bipolar taxa *Carbonea vitellinaria* and *Psilolechia lucida* and the recently reported *Imsharria orangei*, as has been discussed before. Some of these may be representatives of relict biota that persisted through glacial maxima in ice-free refugia [15,122,124]. Although no statistically significant differences were detected in the biogeographical distribution of taxa between the overall nunatak and non-nunatak floras, the proportion of endemic taxa was nonetheless higher in the former (Figure 5). A similar tendency is suggested by the nunatak-exclusive flora, of which nine out of eleven taxa recorded are endemic. While these patterns are descriptive and should be interpreted with caution, they may nonetheless be consistent with the idea that specialization is favored under harsher environmental conditions, and that nunataks may have acted as refugia—and potentially as sources of propagules—for lichen taxa with long-term persistence in Antarctica [15,160].

At higher taxonomic ranks (order and family), compositional differences between nunatak and non-nunatak floras are minimal, indicating that most major lineages are able to colonize nunatak environments. These sites thus host a reduced but not taxonomically truncated assemblage relative to other Antarctic habitats, with turnover occurring primarily at the species level. This phenomenon is well illustrated in the maritime Antarctic region, where only 25.5% of the species recorded in non-nunatak areas are shared with nunataks. However, when viewed from the nunataks’ perspective, this shared subset accounts for 96.0% of their total species count. The comparative lower richness of lichen species and genera on nunataks may primarily reflect ecological and biogeographical constraints, including limited dispersal from non-nunatak areas, reduced tolerance to more severe environmental conditions, and historical contingencies such as past extinctions or restricted colonization opportunities. In addition, taxonomic uncertainty and under-documentation resulting from insufficient collections—affecting markedly inaccessible locations in particular—may partly contribute to the currently low number of species reported from nunatak environments [161]. Although comprehensive floristic surveys have been conducted across Antarctica for decades [5], sampling efforts remain uneven among regions [13,162], at the risk of drawing misleading interpretations of ecological processes [6]. While it may seem unlikely that taxa have been overlooked in coastal and lowland areas given the especially high number of records available for these sites in particular [5,12], supplemental expert-led fieldwork and revision of herbarium material in light of recent taxonomic advances may reveal that some taxa currently considered exclusive to either non-nunatak or nunatak environments are, in fact, shared. Similarly, additional targeted surveys of nunataks may uncover further exclusive taxa and contribute to expanding the currently limited pool of nunatak-restricted lichens.

There appears to be a larger shared species pool between nunatak and surrounding ice-free habitats in continental Antarctica than in the maritime region. This is consistent with the idea that much of the continental landmass supports broadly similar floras due to its overall environmental uniformity resulting from the amalgam of extreme cold, limited moisture availability, and relative scarcity of suitable microhabitats [5]. Under such consistently harsh conditions, only a restricted set of lichens capable of tolerating particularly severe environments can persist. These same taxa occur wherever exposed rock is available and microenvironmental conditions are agreeable across both nunataks and other ice-free landforms alike [163]. In contrast, the maritime Antarctic is macroenvironmentally more heterogeneous, with milder temperatures, greater moisture availability, and more diversified microhabitats across inland and altitudinal gradients. This heterogeneity contributes to stronger differences in species composition between nunatak and non-nunatak areas overall. The pattern highlights a greater influence of nunatak-associated environmental constraints on lichen biodiversity in the maritime Antarctic compared with continental Antarctica.

Crustose lichens dominate both nunatak and non-nunatak floras across the Antarctic continent, although their proportion was slightly higher in nunatak environments. While this difference was not statistically significant, it may nonetheless reflect the influence of the harsher conditions characteristic of nunataks. Strong and abrasive winds can inflict substantial mechanical damage on exposed lichen thalli [109], thereby exerting selective pressure favoring morphotypes capable of withstanding such stress. Crustose lichens, owing to their close adherence to the substrate and limited vertical projection, are markedly more resistant to wind abrasion than other morphotypes [23] and become predominant in the more adverse environments [101,164]. This interpretation is further supported by the observation that all eleven taxa restricted to nunatak environments reported in this study correspond to crustose morphotypes. Consistent with previous surveys of the Hurd Peninsula, nanofruticose thalli were not reported from any of the nunataks considered in the literature synthesis.

Future studies should aim to uncover potential patterns underlying the floristic composition of Antarctic nunataks across regions. Environmental variables (such as ice-free substrata availability, microtopographic complexity, substrate stability and exposure time, edaphic composition, connectivity to neighboring habitats, proximity to the sea, water availability, nutrient enrichment, wind and light exposure, and temperature regimes) ought to be analyzed individually, or rather as an interacting compendium of overlapping influences. However, if present-day nunatak lichen assemblages are the result of long-term survival in situ, establishing direct links between taxonomic composition and contemporary ecological drivers may prove challenging [163]. Scale is also likely to play a role. At local and regional levels, diversity patterns are complex [19,63,124,159,165], such that broad-scale analyses risk overlooking microhabitat heterogeneity and obscuring the very drivers that could explain the current assembly of nunatak lichen populations [6,163,166]. Expert prospections of insufficiently surveyed inland sites—particularly in the continental landmass—are still urgently needed to properly address these knowledge gaps [15]. Such efforts would also benefit studies of genetic structure, gene flow among populations, and taxonomic diversity in nunatak and non-nunatak sites, which are currently constrained by limited sampling of both individual species (poor coverage of a single species among its populations) and different species (lack of representation of many taxa), as well as restricted spatial coverage. Although recent studies have made substantial contributions to the available genetic data for Antarctic lichens [81,98,99,107,127,128,129,167,168,169], additional population-level sampling, including the sequencing of herbarium-preserved material, is required across a broader range of locations—especially nunataks—as well as across a greater diversity of species. Such efforts fall beyond the primary objectives of this study. Nonetheless, we attempted to reconstruct haplotype networks using the available data. The analyses presented here were conducted on a subset of the reported pool of species shared between nunatak and non-nunatak environments, representing no more than 4.5% of the total, and did not yield conclusive results. Addressing this notable gap will require expanding both taxonomic and geographic coverage. This will be essential to determine whether current nunatak lichen communities primarily reflect historical persistence or ongoing dispersal processes.

## 5. Conclusions

This study makes several complementary contributions to the understanding of lichen diversity on Antarctic nunataks. In the first place, we present an initial evaluation of the taxonomic diversity of four nunataks on the Hurd Peninsula. Current floristic richness amounts to 39 confidently identified species, although this number is likely to increase following the taxonomic resolution of currently uncertain records. Botanical surveys provide a foundation for long-term monitoring through resurveying, allowing for the assessment of changes in species presence, abundance, and distribution over time. Given the scarcity of long-term comparative studies in maritime Antarctica—for lichens [170], algae [171], bryophytes [45,171], and vascular plants [172]—the taxonomic inventory gathered here provides new reference sites that may serve as a basis for future observational comparisons. In addition, this study contributes newly generated DNA sequences from previously uncharacterized sites. These sequences expand the availability of reference material from underrepresented locations for future taxonomic, ecological, and phytogeographic research. Continued enrichment of nucleotide databases with lichen sequences from Antarctic ice-free environments will be critical to resolving the taxonomic identity of currently uncertain taxa in our survey. Finally, the information compiled from literature sources and curated databases provides an updated overview of lichen diversity on Antarctic nunataks. The ecological patterns and syntheses presented here provide a framework that may help guide forthcoming investigations into species distributions, regional and broad-scale comparisons of floristic diversity across Antarctic ice-free environments, habitat vulnerability, and the identification of priority areas for biodiversity monitoring and conservation. In this context, these insights may prove increasingly relevant as ongoing climatic shifts may reshape the environmental setting of these isolated Antarctic landscapes [161,162,173,174]. Altogether, this work contributes to establishing a foundation for further floristic, ecological, and molecular studies on nunatak lichens, a still relatively poorly documented component of Antarctica’s terrestrial ecosystems.

## Figures and Tables

**Table 1:** Presence-absence table of lichenized fungi recorded from four nunataks surveyed on the Hurd Peninsula. An “x” indicates the presence of each taxon at a given site. Taxa in bold were identified using molecular analyses followed by morphological revision. Non-bold entries were identified through standard morphological, anatomical, and chemical characterization. Taxa in bold and marked with an asterisk (*) encompass specimens that were identified using either approach.

Taxon Name	Nunatak del Castillo	Napier Peak	Cerro Mirador	MacGregor Peak
*Amandinea* sp.	x			
*Arthopyrenia* sp.		x		
*Austrolecia antarctica* Hertel *	x	x		x
*Austrolecia* aff. *antarctica* Hertel	x			
*Bellemerea alpina* (Sommerf.) Clauzade & Cl. Roux	x		x	x
*Bellemerea subsorediza* (Lynge ex E. Dahl) R. Sant.	x	x		
*Bellemerea* aff. *pullata* (Darb.) Øvstedal	x			
*Bellemerea* aff. *subsorediza* (Lynge ex E. Dahl) R. Sant.	x	x		
***Buellia* aff. *russa* (Hue) Darb.**		x		
*Buellia* aff. *soredians* Filson	x			
*Buellia* sp.	x			
*Carbonea vorticosa* (Flörke) Hertel	x			
*Carbonea* aff. *assentiens* (Nyl.) Hertel	x			
*Carbonea* aff. *vorticosa* (Flörke) Hertel	x			
*Carbonea* sp.	x			
*Fuscidea* sp.	x			
*Himantormia lugubris* (Hue) I.M. Lamb	x	x	x	x
***Imsharria orangei* Fryday & U. Rupr.**	x	x		
*Lecanora griseosorediata* Øvstedal		x		x
*Lecanora physciella* (Darb.) Hertel *	x	x	x	x
***Lecanora physciella* var. *sorediata* Øvstedal**		x		
*Lecanora polytropa* (Hoffm.) Rabenh.	x	x		
*Lecanora* aff. *griseosorediata* Øvstedal *	x	x		
*Lecanora* aff. *physciella* (Darb.) Hertel	x			
***Lecanoraceae* sp.**	x		x	
*Lecidea atrobrunnea* (DC.) Schaer.	x			x
*Lecidea* aff. *atrobrunnea* (DC.) Schaer.	x			
***Lecidea* sp.**	x	x		
***Lecideaceae* sp.**	x			
*Lecidella siplei* (C.W. Dodge & G.E. Baker) May. Inoue				x
*Lecidella wulfenii* (Ach.) Körb.	x			
***Lecidella* sp.**	x	x		x
*Lepra excludens* (Nyl.) Hafellner	x		x	x
***Lepra* aff. *corallophora* (Vain.) Hafellner**			x	
***Lepra* sp.**	x			
***Lepraria caerulescens* (Hue) Botnen & Øvstedal**	x			
*Ochrolechia frigida* (Sw.) Lynge	x			
*Ochrolechia parella* (L.) A. Massal.				x
*Placopsis antarctica* D.J. Galloway, R.I.L. Sm. & Quilhot	x		x	x
*Placopsis contortuplicata* I.M. Lamb *	x			
*Poeltidea perusta* (Nyl.) Hertel & Hafellner *	x	x		x
***Poeltidea* sp.**	x			
*Polyozosia* aff. *dispersa* (Pers.) S.Y. Kondr., Lőkös & Farkas	x			
*Pseudephebe minuscula* (Nyl. ex Arnold) Brodo & D. Hawksw.	x		x	
*Psoroma hypnorum* (Vahl) Gray	x			
***Psoroma* aff. *dichroum* (Hook. f. & Taylor) P.M. Jørg.**	x			
*Rhizocarpon badioatrum* (Flörke ex Spreng.) Th. Fr.		x		
*Rhizocarpon geographicum* (L.) DC. *	x	x	x	x
*Rhizocarpon grande* (Flörke ex Flot.) Arnold	x			x
***Rhizocarpon* aff. *badioatrum* (Flörke ex Spreng.)** Th. Fr. *		x		
*Rinodina* aff. *olivaceobrunnea* C.W. Dodge & G.E. Baker	x			
*Sporastatia testudinea* (Ach.) A. Massal. *	x	x		
*Sporastatia* aff. *polyspora* (Nyl.) Grummann	x			
*Staurothele* sp.	x			
*Stereocaulon alpinum* Laurer	x			
*Stereocaulon antarcticum* Vain.	x			
*Stereocaulon glabrum* (Müll. Arg.) Vain. *	x			x
***Stereocaulon vesuvianum* Pers.**		x		
*Stereocaulon* sp.	x			
*Tephromela atra* (Huds.) Hafellner	x	x		
*Thelenella antarctica* (I.M. Lamb) O.E. Erikss.		x		
*Thelenella* aff. *mawsonii* (C.W. Dodge) H. Mayrhofer & P.M. McCarthy	x			
*Tremolecia atrata* (Ach.) Hertel *	x	x		
*Umbilicaria africana* (Jatta) Krog & Swinscow	x	x		
*Umbilicaria cristata* C.W. Dodge & G.E. Baker	x			
*Umbilicaria decussata* (Vill.) Zahlbr. *	x			
*Umbilicaria krascheninnikovii* (Savicz) Zahlbr.	x			
*Usnea antarctica* Du Rietz	x			
*Usnea aurantiacoatra* (Jacq.) Bory	x	x	x	x
unidentified lecideoid morphotype	x	x	x	
unidentified sorediate morphotype	x	x		

## Data Availability

The data supporting the findings of the study are available within the article and its Supplementary Materials. The specimens collected for this study have been deposited in the MAF Herbarium at the Complutense University of Madrid for long-term preservation (voucher numbers MAF Lich 25631 to MAF Lich 25883 and MAF Lich 25889 to MAF Lich 25896). Newly generated sequences have been submitted to GenBank, and a detailed list of specimens and their corresponding accession numbers is provided in Table S2.

## Supplementary Materials

The following supporting information can be downloaded at https://www.mdpi.com/article/10.3390/jof12050314/s1. Figure S1: Representative views of the nunataks surveyed in this study. (**a**) Nunatak del Castillo (red arrow), followed by Napier Peak (purple arrow), Cerro Mirador (orange arrow), and Moores Peak (green arrow), with the Tangra Mountains in the background. (**b**) Southern and eastern sides of Nunatak del Castillo, viewed from Napier Peak, with Willan Nunatak further to the right. (**c**) Part of a sampled outcrop on Napier Peak (bottom left side of the picture) overlooking False Bay, with the terminus of Huntress Glacier visible. (**d**) Outcrop on Cerro Mirador, with False Bay beyond. (**e**) View of the southwestern flank of MacGregor Peak, looking toward Miers Bluff. (**f**) Outcrop on the summit of MacGregor Peak. (**g**) Vertical rock face at Nunatak del Castillo, photographed looking upwards, showing lichen colonization. (**h**) *Placopsis contortuplicata* at Nunatak del Castillo. Photographs by Leopoldo G. Sancho (b, c, f, g, and h) and Sergi Ricart Ibars (a, d, and e); Figure S2: Phylogenetic tree of the lichenized fungi retrieved in the survey and related taxa, inferred using maximum likelihood based on the nrITS marker. Branch support values are displayed above the corresponding branches and are given as SH-aLRT/UFBoot. Only branches supported by both SH-aLRT ≥ 80% and UFBoot ≥ 95% bear support values and are depicted in bold. Branch tips are labelled with GenBank accession numbers (blue) and taxon names (black). Newly generated sequences are printed in bold. Family affiliations are indicated to the right of the tree tips. *Leotia lubrica* and *Neodermea acerina* were used as outgroups. The scale bar represents 0.1 substitutions per site; Figure S3: Bar plot showing the number of taxa per rank in both nunatak and non-nunatak environments, relative to the total recorded taxa from each area (Non-Nunatak: n = 559, Nunatak: n = 255); Figure S4: Bar plot depicting the biogeographic origin of species, subspecies, and varieties in nunataks from the continental (green) and maritime regions (yellow); Figure S5: Bar plot depicting the biogeographic origin of species and varieties in Hurd Peninsula non-nunatak (black) and nunatak areas (red). The nunataks considered include the four surveyed in the present study (Nunatak del Castillo, Napier Peak, Cerro Mirador, and MacGregor Peak) as well as Moores Peak; Figure S6: Statistical parsimony networks for the following species based on the nrITS marker: *Acarospora gwynii*, *Austrolecia antarctica*, *Lecanora fuscobrunnea*, *L. physciella*, *Lecidea cancriformis*, *Lecidella siplei*, *Pseudephebe minuscula*, and *Usnea lambii*. Samples are grouped by region and habitat (see Table S5): non-nunatak environments in continental Antarctica (light blue), nunatak environments in continental Antarctica (dark blue), non-nunatak environments in maritime Antarctica (light orange), and nunatak environments in maritime Antarctica (dark orange). The number of samples sharing each haplotype is indicated in italics below the corresponding haplotype label. Within each haplotype network, circle size is proportional to haplotype frequency. Circle sizes are not directly comparable among networks due to differences in sequence abundance across species. Mutational steps are represented by hatch marks along the connections between haplotypes, with each mark corresponding to a single nucleotide difference; Table S1: Top BLAST matches in the UNITE database for each nrITS sequence generated in this study. For each newly generated sequence, the ten highest-scoring matches are listed with the following information: GenBank accession number, sequence ID, and taxon name of the newly generated sequence; GenBank accession number of the matched reference sequences and their corresponding taxon names, Species Hypothesis (SH) identifier at 0.5%, 1.0%, and 1.5% distance thresholds; alignment score, E-value, percent identity, coverage, and number of mismatches; Table S2: GenBank accession numbers for the nrITS sequences used to construct the phylogenetic trees featured in Figure 3 and Figure S2. Species names were updated, where necessary, to reflect current nomenclature according to the Catalogue of Life and Species Fungorum databases. The original names listed in GenBank are given in the column called ‘Original taxonomic assignment’. ‘Direct submission’ refers to sequences deposited in GenBank that are not associated with a peer-reviewed publication; Table S3: List of lichenized fungi recorded from four nunataks surveyed on the Hurd Peninsula (Livingston Island, South Shetland Islands). The family, order, biogeographic distribution, morphotype, and reproductive strategy are given for each taxon. Authorships are listed in Table 1; Table S4: Summary of reference sites included for comparison between Antarctic nunatak and non-nunatak environments. For each site, the corresponding Antarctic Conservation Biogeographic Region (ACBR; Terauds & Lee, 2016), region (continental or maritime), habitat (nunatak or non-nunatak), and geographic coordinates (in decimal degrees) are provided. For sites encompassing multiple sampling locations, ranges of latitudinal (northernmost to southernmost) and longitudinal (easternmost to westernmost) coordinates are reported; Table S5: GenBank accession numbers and associated collection data for the nrITS sequences used to construct the haplotype networks. The corresponding haplotype, region (continental or maritime), habitat (nunatak or non-nunatak), and ACBR are provided for each sequence. ‘Direct submission’ refers to sequences deposited in GenBank that are not associated with a peer-reviewed publication.

## Appendix A. Surveyed Nunataks in Livingston Island

Sampling was carried out by Ana Aramburu, José Raggio, and Leopoldo G. Sancho in Nunatak del Castillo, by José Raggio and Leopoldo G. Sancho in Napier Peak and Cerro Mirador, and by Leopoldo G. Sancho exclusively in MacGregor Peak. Determinations were made by Leopoldo G. Sancho.

**Table A1:** Characteristics of the surveyed nunataks in Livingston Island. Geographic coordinates are provided in decimal degrees.

Nunatak	Latitude	Longitude	Altitude (m a.s.l.)	Site Description	Sampling Date
Nunatak del Castillo	−62.65447	−60.29406	449	The site is a conspicuous rocky outcrop with extensive ice-free surfaces spanning a wide range of orientations, slopes, and elevations. It includes steep southern and northern faces and a broad, flat summit with a sharp vertical drop.	27 January 2022, 1 February 2022, 12 January 2023, 14 January 2023
Napier Peak	−62.67167	−60.32500	340	Located approximately 2.5 km southwest of Nunatak del Castillo, this area rises to the west of Huntress Glacier and consists of several closely spaced peaks and ridges protruding slightly through the surrounding ice.	17 January 2023
Cerro Mirador	−62.68150	−60.33972	307	Situated in the southeastern part of Hurd Peninsula, overlooking False Bay and about 1.35 km south-southwest of Napier Peak, Cerro Mirador (also known as Mirador Hill) is a rounded and broad rocky hill with relatively extensive exposed surfaces.	16 January 2023
MacGregor Peak	−62.69667	−60.39306	407	The southernmost site in the study area lies near the southeastern end of Hurd Peninsula, facing False Bay and approximately 3.5 km from Cerro Mirador. It consists primarily of heavily frost-fractured shales.	26 January 2023

## Appendix B. PCR Amplification Protocol

PCR reactions were carried out in a total volume of 25 μL, containing 5 μL of DNA from the second elution of the extraction, 12.5 μL of AmpliTools DNA Master Mix (Biotools, Madrid, Spain), 1.5 μL of each primer (10 μM), and 4.5 μL of sterile ultrapure water produced using a Milli-Q system equipped with a Millipak^®^ Express 40 filter (Merck, Darmstadt, Germany). Amplifications were performed in an automatic thermocycler (Bioer XP Cycler, Hangzhou, China). The PCR protocol followed a touchdown strategy, which began with an initial denaturation at 95 °C for five minutes, followed by three cycles with progressively decreasing annealing temperatures: 67 °C, 64 °C, and 56 °C. Each of these initial cycles included denaturation at 95 °C for 30 s, annealing at the specified temperature for 30 s, and extension at 72 °C for one minute. Following the touchdown phase, 30 amplification cycles were performed using the same denaturation and extension conditions, with a fixed annealing temperature of 55 °C. A final extension step was carried out at 72 °C for 10 min. PCR products were visualized by electrophoresis on a 1% agarose gel using SYBR Safe DNA (Life Technologies Corporations, Carlsbad, CA, USA) and purified using the SpeedTools PCR Clean-Up kit (Biotools). The purified amplicons were then sequenced in both directions by Macrogen Spain (https://www.macrogen.com/) using Sanger sequencing. Sequencing reactions were conducted on a DNA Engine Tetrad 2 Peltier Thermal Cycler (BIO-RAD, Hercules, CA, USA), with the ABI BigDye^®^ Terminator v3.1 Cycle Sequencing Kit (Applied Biosystems, Foster City, CA, USA). Products were purified according to the manufacturer’s protocol and electrophoresed on an ABI PRISM 3730XL DNA Analyzer (Applied Biosystems).

## Appendix C. Sequence Alignment

The forward and reverse sequences of each sample were paired-end assembled to generate a consensus sequence using the command De Novo Assembler, Highest Sensitivity and without enabling sequence trimming. Sequences that failed to assemble under these settings were reprocessed using paired-end assembly with trimming allowed. For all samples for which paired-end assembly was unsuccessful, single-end sequences were considered. Electropherograms from both paired-end and single-end sequences were examined, and manual corrections were made where necessary. Base calls were considered unresolvable when overlapping secondary peaks exceeded 25% of the maximal peak’s height. Sequences with more than 1% ambiguous calls with respect to total sequence length were excluded from the final dataset.

## Appendix D. Checklist Creation

To broaden the scope of the lichen occurrence dataset compiled for nunataks and to assess the distinctiveness of nunatak flora relative to overall Antarctic lichen biodiversity, we supplemented it with species-occurrence records from non-nunatak environments derived from published floristic checklists and existing databases, including the Ice-free Antarctic Biodiversity Database [12] and TerrANTALife 1.0 [175].

Firstly, an exhaustive review of floristic data from the published literature was conducted to incorporate records of taxa reported from non-nunatak locations into the existing dataset (Table S4). Non-nunatak environments were defined as areas that are generally (but not exclusively) low-elevation and predominantly coastal, encompassing a broad range of orographic features under varying degrees of maritime influence, including manured rocks and stones in the vicinity of bird rookeries, bluffs, coastal cliffs, assemblages of low hills and valleys, moraine drifts, scree slopes, cirques, lakes margins, riverside terrains, and sheltered or exposed rocky promontories and mounts that do not fall within the nunatak category because they are not encircled by permanent glacial ice. Inland oases with distinct environmental regimes, such as Amery Oasis (here referring specifically to the Radok Lake area, including the nearby Clemence Massif, considered a separate oasis further inland within the Lambert Glacier by Andreev [20]), Bunger Hills, Lake Untersee Oasis, and Schirmacher Oasis were also incorporated as non-nunatak sites.

To ensure representativeness, we selected floristic works that, taken together, covered the broadest possible latitudinal range, spanning both the maritime region (which comprises the west side of the Antarctic Peninsula north of about 72° S and the adjacent islands south of 60° S) and the continental region (including the main continental landmass, the eastern and southernmost part of the Antarctic Peninsula, and its ascribed offshore islands south of 60° S) [101]. Accordingly, sites from both the macroenvironment and microenvironmental zones (north and south of ~72° S, respectively) were included [163]. Floristic data were drawn from localities distributed across the major Antarctic Conservation Biogeographic Regions (ACBRs; [9]), although ACBRs 13 and 14 (Adelie Land and Ellsworth Land, respectively) remained unrepresented. Coincidentally, these two regions show the lowest number of records in the Ice-free Antarctic Biodiversity Database, alongside the Ellsworth Mountains (ACBR 11) [12]. The latter has also been identified as containing the smallest area of vegetation detected based on satellite imagery and spectral indices [10].

The Ice-free Antarctic Biodiversity Database was merged with the refined checklist, with the constraint that a maximum of two records per taxon were to be retained for non-nunatak areas (one for the maritime region and one for the continental region, when applicable). In contrast, each record reported from a given nunatak or group of nunataks was retained as a separate entry, regardless of region. The geographical coordinates provided for each entry of the Ice-free Antarctic Biodiversity Database were used to assist in determining whether specimens were sampled from nunatak or non-nunatak environments, following the criteria established to distinguish between these two site types detailed in Section 2.4 of the manuscript. Subsequently, the resulting checklist was cross-linked with TerrANTALife 1.0 due to it having been revised by expert lichenologists. All records from TerrANTALife 1.0 not listed in the Ice-free Antarctic Biodiversity Database were kept in our dataset. This approach improved the completeness of the catalogue of taxa identified in both nunatak and non-nunatak areas.

The final checklist was manually curated to avoid the retention of misidentifications, correct spelling errors, and resolve synonymities. The revision of Antarctic lichens by Castello and Nimis [176] was consulted to aid in synonymizing taxa listed in earlier floras. A detailed list of nomenclatural amendments is provided in Appendix E.

The nomenclature of the final dataset was updated with the Catalogue of Life database (version from 16 November 2025), and both authorship and higher-level taxonomic classifications were verified. Taxa that were not listed in the Catalogue of Life were checked in Species Fungorum (https://www.speciesfungorum.org/, accessed on 9 December 2025), MycoBank [177] (https://www.mycobank.org/, accessed on 9 December 2025), and the Consortium of Lichen Herbaria (https://lichenportal.org/portal/, accessed on 9 December 2025). Species absent from the taxonomic databases consulted were removed [12], as were those not assigned to any taxonomic category in the source literature (e.g., “pustulate crustose lichen”, “several unidentified crustose taxa”, “unidentified sterile lichens”). Likewise, informal or author-specific labels used in some of the published literature consulted that did not correspond to formally recognized taxonomic ranks (e.g., *Aspicilia* sp. 1 and *Aspicilia* sp. 2, or *Buellia* sp. greyish and *Buellia* sp. yellowish, among others) were not treated as distinct species. Such records were retained only at the taxonomic level explicitly supported by the available information, as assigning further specificity would involve unsupported assumptions.

## Appendix E. Information on Nomenclatural Amendments

Several nomenclatural updates were applied in accordance with recent taxonomic revisions. Specimens formerly reported as *Bacidia* sp. A in maritime Antarctica or mistakenly listed as *B. trachona* from King George Island and Livingston Island were renamed *B. chrysocolla* [178]. *Bacidina subfuscula* was chosen over *Lecania subfuscula* (as listed in the Catalogue of Life) following the most recent work by Ekman [179]. According to Søchting et al. [180], material collected from the Antarctic Peninsula and originally published as *Caloplaca* aff. *anchon-phoeniceon* in Søchting and Øvstedal [181] and Øvstedal and Lewis Smith [8] was transferred to *Catenarina desolata*. In turn, records of *Caloplaca sublobulata* (synonym of *Gondwania sublobulata*) were reassigned to *Gondwania joannae* [182]. The name *Gondwania regalis* was prioritized over *Polycauliona regalis* (as listed in the Catalogue of Life) based on the work by Søchting et al. [182]. Following Passo et al. [183], *Cetraria subscutata* was treated as a synonym of *Nephromopsis chlorophylla*. *Flavoplaca citrina* was reassigned to either *Austroplaca darbishirei* or *A. soropelta* in accordance with the information provided in Søchting and Castello [184]. Records of *Hueidea cerussata*, *H. coeruleofrigida*, *H. grisea*, *H. sorediata*, and *H. vaczii* were retained even though they do not yet appear on the Catalogue of Life database [185]. *Lecanora physciella* var. *sorediata* (sensu Øvstedal & Lewis Smith [8]) was accepted and therefore not treated as a synonym of *L. physciella*. The latest work where it is cited as an independent taxon is that of Ochyra et al. [63]. *Lecidella protracta* was transferred to *Palicella xantholeuca* following Fryday et al. [105]. Based on the work by Barcenas-Peña et al. [186], *Lepraria caerulescens* (Hue) Botnen & Øvstedal was regarded as a separate species from *L. alpina* (B.de Lesd.) Tretiach & Baruffo. All records of *Lepraria cacuminum* (A.Massal.), Kümmerl. & Leuckert, *L. cacuminum* (A.Massal.) Lohtander, and *Leproloma cacuminum* (A.Massal.) J. R. Laundon were consolidated under *Lepraria alpina* (B. de Lesd.) Tretiach & Baruffo [187,188,189]. According to Jørgensen et al. [190], *Massalongia carnosa* records were transferred to *M. patagonica*, as the former species is restricted to the Northern Hemisphere. *Massalongia intricata* from Livingston Island, listed in Søchting et al. [44], was reassigned to *Austrella isidioidea* [191]. All other records of *M. intricata* were transferred to *Steinera intricata* [191]. Based on Fryday and Hertel [192], *Notolecidea ochyrae* specimens cited in Søchting et al. [44] were renamed *Poeltiaria coppinsiana*. Although the Catalogue of Life claims that *Phyllisciella marionensis* and *Zahlbrucknerella marionensis* are synonymous, we have considered them both as distinct entities following the work by Prieto et al. [193]. Records of *Placopsis parellina* were discarded, and collections referring to sorediate specimens were instead assigned to *P. antarctica* [194]. Reports of *Pseudephebe pubescens* were reclassified as *P. minuscula* [107]. Finally, *Usnea sphacelata* was replaced by *U. lambii* [102,103].
