# Supplementary material for: Islands of Biodiversity: Characterization of Lichen Flora in Antarctic Nunataks
Source: J Fungi (Basel). 2026 Apr 24;12(5):314. doi: 10.3390/jof12050314 (PMC13207803; doi:10.3390/jof12050314)
Supplement: Supplementary file 1 [file jof-12-00314-s001.zip › sup-mat_JoF_AAC.pdf]

Supplementary Materials

Islands of biodiversity: characterization of lichen flora in Antarctic nunataks

Ana Aramburu, Núria Beltran-Sanz, José Raggio, Pradeep K. Divakar, Ana Pintado, Asunción de los Ríos, Leopoldo G. Sancho

Figure S1. Representative views of the nunataks surveyed in this study. (**a**) Nunatak del Castillo (red arrow), followed by Napier Peak (purple arrow), Cerro Mirador (orange arrow), and Moores Peak (green arrow), with the Tangra Mountains in the background. (**b**) Southern and eastern sides of Nunatak del Castillo, viewed from Napier Peak, with Willan Nunatak further to the right. (**c**) Part of a sampled outcrop on Napier Peak (bottom left side of the picture) overlooking False Bay, with the terminus of Huntress Glacier visible. (**d**) Outcrop on Cerro Mirador, with False Bay beyond. (**e**) View of the southwestern flank of MacGregor Peak, looking toward Miers Bluff. (**f**) Outcrop on the summit of MacGregor Peak. (**g**) Vertical rock face at Nunatak del Castillo, photographed looking upwards, showing lichen colonization. (**h**) *Placopsis contortuplicata* at Nunatak del Castillo. Photographs by Leopoldo G. Sancho (b, c, f, g, and h) and Sergi Ricart Ibars (a, d, and e).

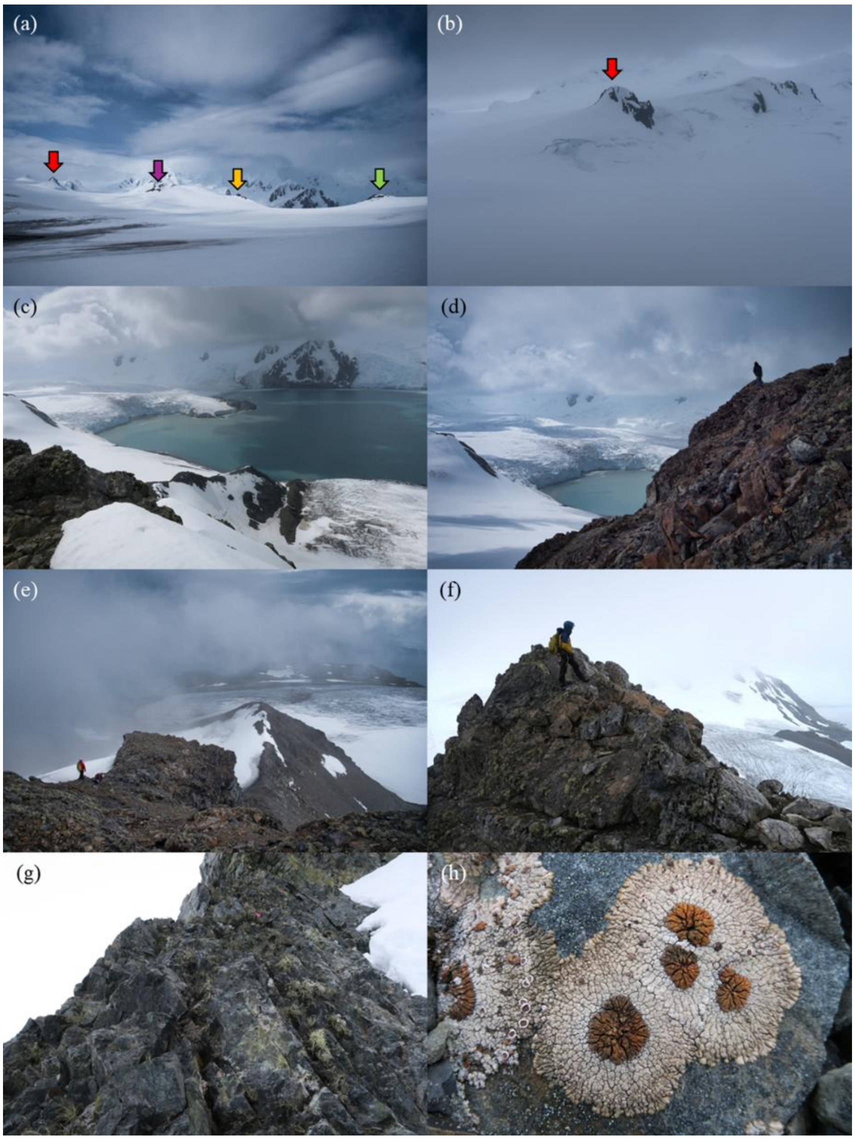

Figure S2. Phylogenetic tree of the lichenized fungi retrieved in the survey and related taxa, inferred using maximum likelihood based on the nrITS marker. Branch support values are displayed above the corresponding branches and are given as SH-aLRT/UFBoot. Only branches supported by both SH-aLRT ≥ 80% and UFBoot ≥ 95% bear support values and are depicted in bold. Branch tips are labelled with GenBank accession numbers (blue) and taxon names (black). Newly generated sequences are printed in bold. Family affiliations are indicated to the right of the tree tips. *Leotia lubrica* and *Neodermea acerina* were used as outgroups. The scale bar represents 0.1 substitutions per site.

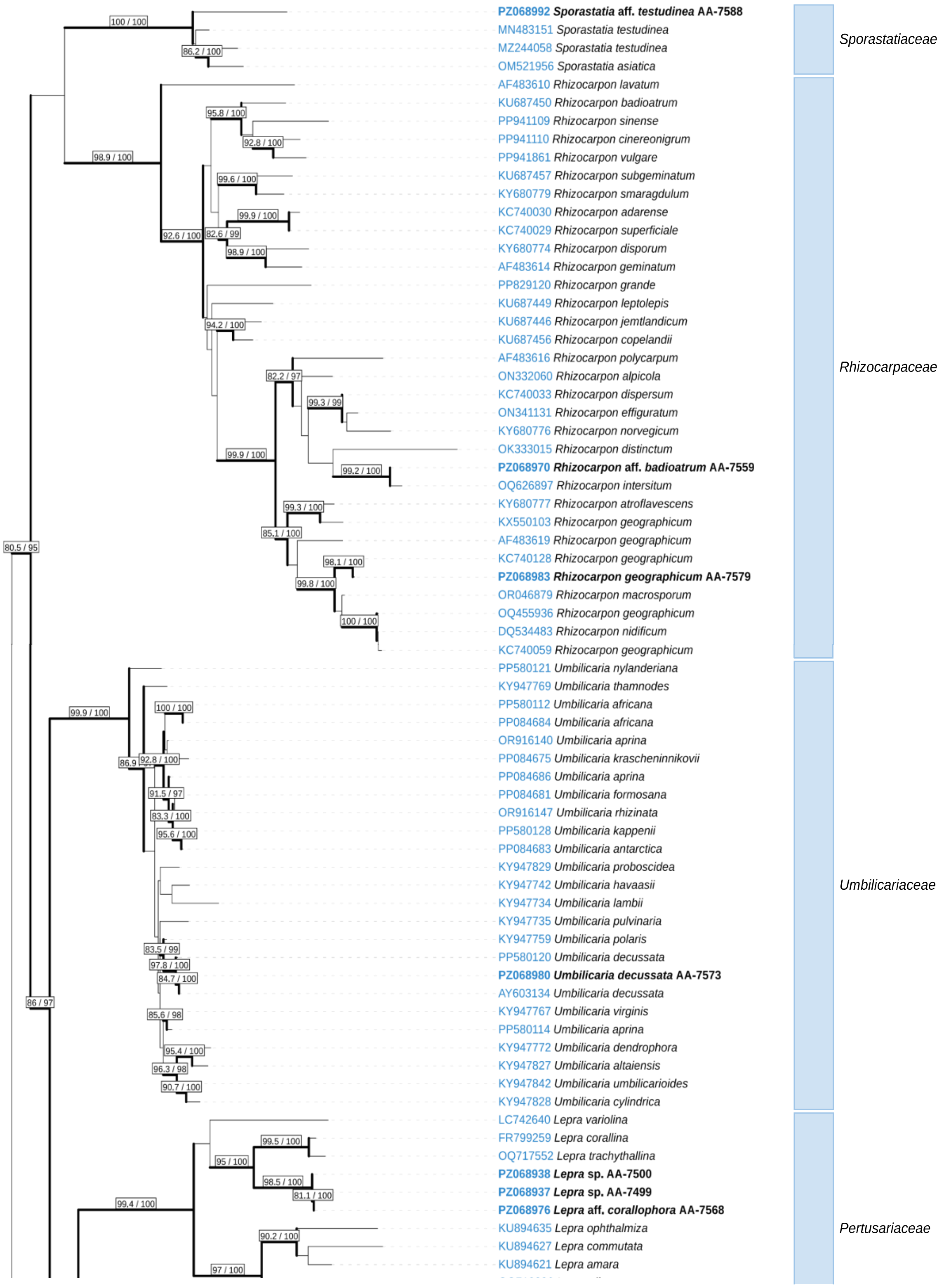

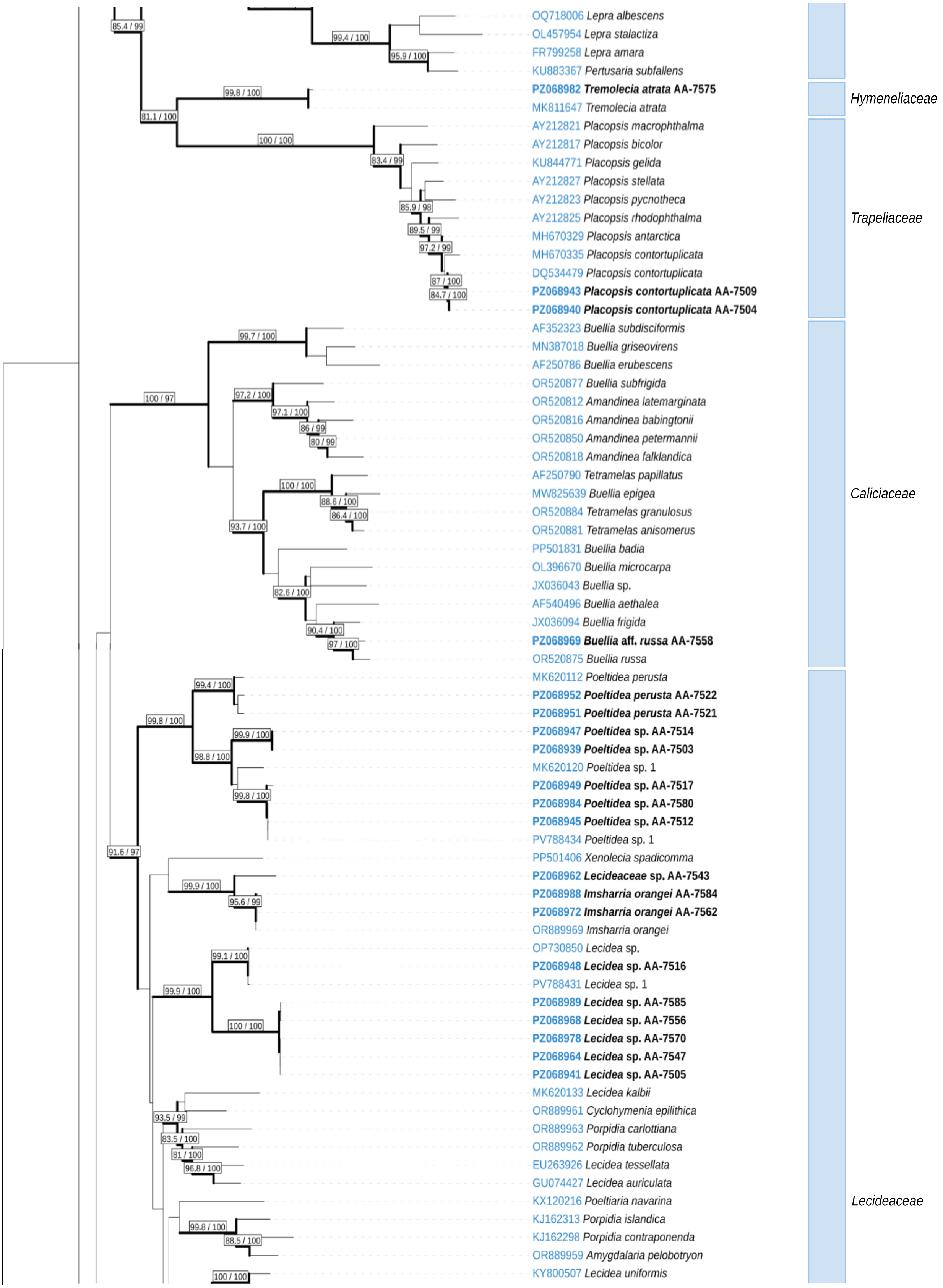

OQ718006 Lepra albescens
OL457954 Lepra stalactiza
FR799258 Lepra amara
KU883367 Pertusaria subfallens
PZ068982 Tremolecia atrata AA-7575
MK811647 Tremolecia atrata
AY212821 Placopsis macrophthalma
AY212817 Placopsis bicolor
KU844771 Placopsis gelida
AY212827 Placopsis stellata
AY212823 Placopsis pycnotheca
AY212825 Placopsis rhodophthalma
MH670329 Placopsis antarctica
MH670335 Placopsis contortuplicata
DQ534479 Placopsis contortuplicata
PZ068943 Placopsis contortuplicata AA-7509
PZ068940 Placopsis contortuplicata AA-7504
AF352323 Buellia subdisciformis
MN387018 Buellia griseovirens
AF250786 Buellia erubescens
OR520877 Buellia subfrigida
OR520812 Amandinea latemarginata
OR520816 Amandinea babingtonii
OR520850 Amandinea petermannii
OR520818 Amandinea falklandica
AF250790 Tetramelas papillatus
MW825639 Buellia epigea
OR520884 Tetramelas granulosus
OR520881 Tetramelas anisomerus
PP501831 Buellia badia
OL396670 Buellia microcarpa
JX036043 Buellia sp.
AF540496 Buellia aethalea
JX036094 Buellia frigida
PZ068969 Buellia aff. russa AA-7558
OR520875 Buellia russa
MK620112 Poeltidea perusta
PZ068952 Poeltidea perusta AA-7522
PZ068951 Poeltidea perusta AA-7521
PZ068947 Poeltidea sp. AA-7514
PZ068939 Poeltidea sp. AA-7503
MK620120 Poeltidea sp. 1
PZ068949 Poeltidea sp. AA-7517
PZ068984 Poeltidea sp. AA-7580
PZ068945 Poeltidea sp. AA-7512
PV788434 Poeltidea sp. 1
PP501406 Xenolecia spadicomma
PZ068962 Lecideaceae sp. AA-7543
PZ068988 Imsharria orangei AA-7584
PZ068972 Imsharria orangei AA-7562
OR889969 Imsharria orangei
OP730850 Lecidea sp.
PZ068948 Lecidea sp. AA-7516
PV788431 Lecidea sp. 1
PZ068989 Lecidea sp. AA-7585
PZ068968 Lecidea sp. AA-7556
PZ068978 Lecidea sp. AA-7570
PZ068964 Lecidea sp. AA-7547
PZ068941 Lecidea sp. AA-7505
MK620133 Lecidea kalbii
OR889961 Cyclohymenia epilithica
OR889963 Porpidia carlottiana
OR889962 Porpidia tuberculosa
EU263926 Lecidea tessellata
GU074427 Lecidea auriculata
KX120216 Poeltiaria navarina
KJ162313 Porpidia islandica
KJ162298 Porpidia contraponenda
OR889959 Amygdalaria pelobotryon
KY800507 Lecidea uniformis
Hymeneliaceae
Trapeliaceae
Caliciaceae
Lecideaceae

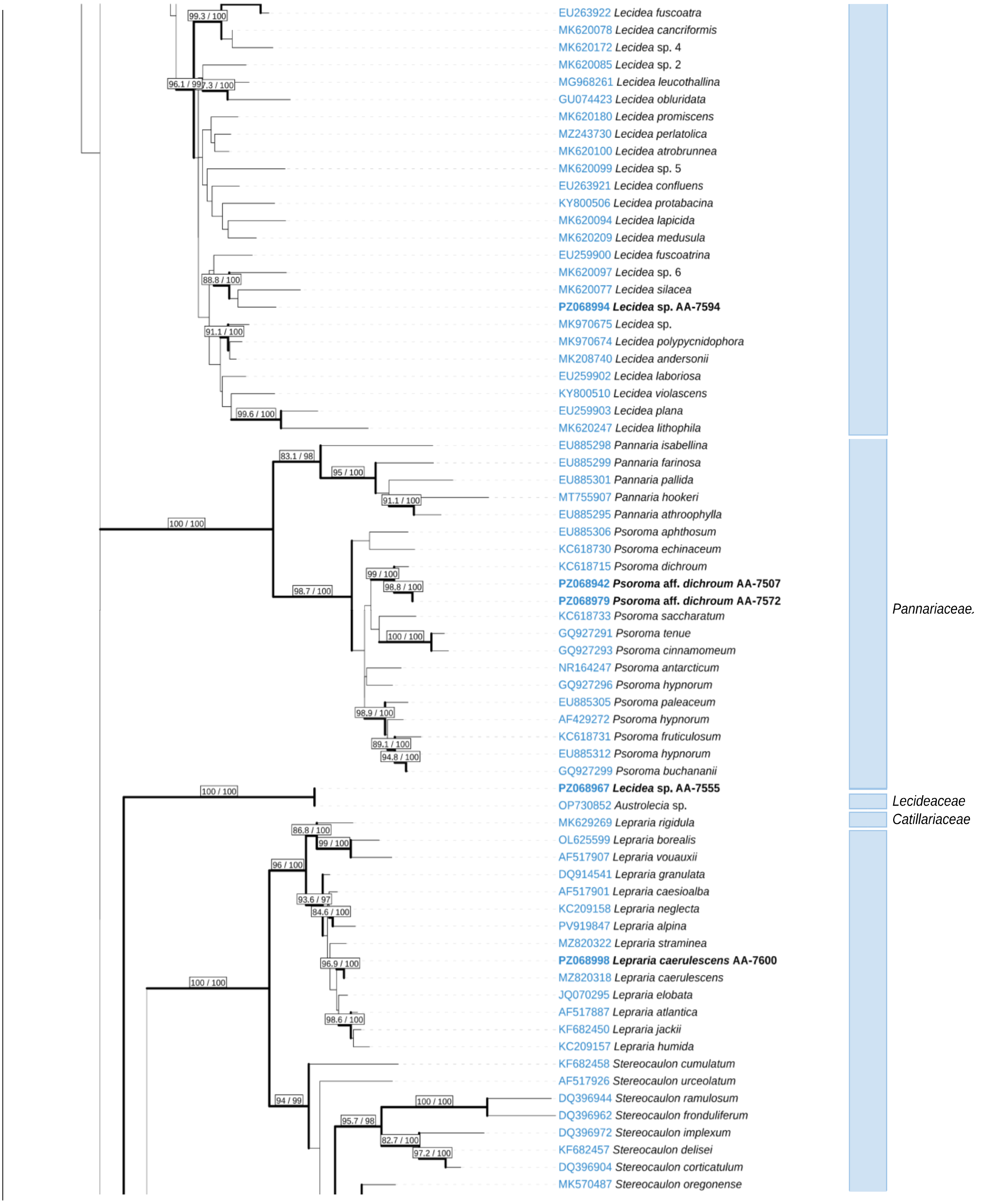
EU263922 Lecidea fuscoatra
MK620078 Lecidea cancriformis
MK620172 Lecidea sp. 4
MK620085 Lecidea sp. 2
MG968261 Lecidea leucothallina
GU074423 Lecidea obluridata
MK620180 Lecidea promiscens
MZ243730 Lecidea perlatolica
MK620100 Lecidea atrobrunnea
MK620099 Lecidea sp. 5
EU263921 Lecidea confluens
KY800506 Lecidea protabacina
MK620094 Lecidea lapicida
MK620209 Lecidea medusula
EU259900 Lecidea fuscoatrina
MK620097 Lecidea sp. 6
MK620077 Lecidea silacea
PZ068994 Lecidea sp. AA-7594
MK970675 Lecidea sp.
MK970674 Lecidea polypycnidophora
MK208740 Lecidea andersonii
EU259902 Lecidea laboriosa
KY800510 Lecidea violascens
EU259903 Lecidea plana
MK620247 Lecidea lithophila
EU885298 Pannaria isabellina
EU885299 Pannaria farinosa
EU885301 Pannaria pallida
MT755907 Pannaria hookeri
EU885295 Pannaria athroophylla
EU885306 Psoroma aphthosum
KC618730 Psoroma echinaceum
KC618715 Psoroma dichroum
PZ068942 Psoroma aff. dichroum AA-7507
PZ068979 Psoroma aff. dichroum AA-7572
KC618733 Psoroma saccharatum
GQ927291 Psoroma tenue
GQ927293 Psoroma cinnamomeum
NR164247 Psoroma antarcticum
GQ927296 Psoroma hypnorum
EU885305 Psoroma paleaceum
AF429272 Psoroma hypnorum
KC618731 Psoroma fruticulosum
EU885312 Psoroma hypnorum
GQ927299 Psoroma buchananii
PZ068967 Lecidea sp. AA-7555
OP730852 Austrolecia sp.
MK629269 Lepraria rigidula
OL625599 Lepraria borealis
AF517907 Lepraria vouauxii
DQ914541 Lepraria granulata
AF517901 Lepraria caesioalba
KC209158 Lepraria neglecta
PV919847 Lepraria alpina
MZ820322 Lepraria straminea
PZ068998 Lepraria caerulescens AA-7600
MZ820318 Lepraria caerulescens
JQ070295 Lepraria elobata
AF517887 Lepraria atlantica
KF682450 Lepraria jackii
KC209157 Lepraria humida
KF682458 Stereocaulon cumulatum
AF517926 Stereocaulon urceolatum
DQ396944 Stereocaulon ramulosum
DQ396962 Stereocaulon fronduliferum
DQ396972 Stereocaulon implexum
KF682457 Stereocaulon delisei
DQ396904 Stereocaulon corticatulum
MK570487 Stereocaulon oregonense
Pannariaceae
Lecideaceae
Catillariaceae

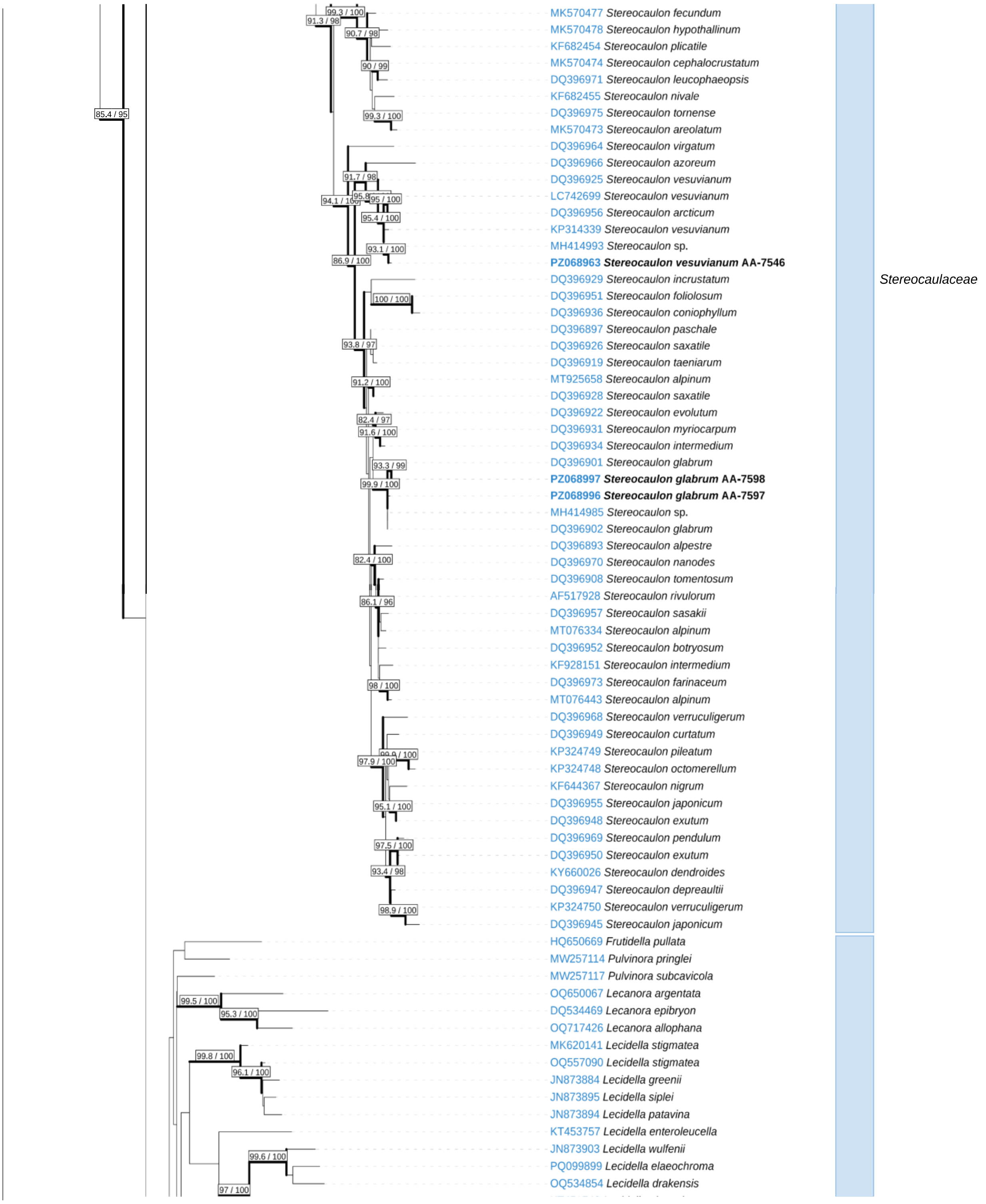

MK570477 Stereocaulon fecundum
MK570478 Stereocaulon hypothallinum
KF682454 Stereocaulon plicatile
MK570474 Stereocaulon cephalocrustatum
DQ396971 Stereocaulon leucophaeopsis
KF682455 Stereocaulon nivale
DQ396975 Stereocaulon tornense
MK570473 Stereocaulon areolatum
DQ396964 Stereocaulon virgatum
DQ396966 Stereocaulon azoreum
DQ396925 Stereocaulon vesuvianum
LC742699 Stereocaulon vesuvianum
DQ396956 Stereocaulon arcticum
KP314339 Stereocaulon vesuvianum
MH414993 Stereocaulon sp.
PZ068963 Stereocaulon vesuvianum AA-7546
DQ396929 Stereocaulon incrustatum
DQ396951 Stereocaulon foliolosum
DQ396936 Stereocaulon coniophyllum
DQ396897 Stereocaulon paschale
DQ396926 Stereocaulon saxatile
DQ396919 Stereocaulon taeniarum
MT925658 Stereocaulon alpinum
DQ396928 Stereocaulon saxatile
DQ396922 Stereocaulon evolutum
DQ396931 Stereocaulon myriocarpum
DQ396934 Stereocaulon intermedium
DQ396901 Stereocaulon glabrum
PZ068997 Stereocaulon glabrum AA-7598
PZ068996 Stereocaulon glabrum AA-7597
MH414985 Stereocaulon sp.
DQ396902 Stereocaulon glabrum
DQ396893 Stereocaulon alpestre
DQ396970 Stereocaulon nanodes
DQ396908 Stereocaulon tomentosum
AF517928 Stereocaulon rivulorum
DQ396957 Stereocaulon sasakii
MT076334 Stereocaulon alpinum
DQ396952 Stereocaulon botryosum
KF928151 Stereocaulon intermedium
DQ396973 Stereocaulon farinaceum
MT076443 Stereocaulon alpinum
DQ396968 Stereocaulon verruculigerum
DQ396949 Stereocaulon curtatum
KP324749 Stereocaulon pileatum
KP324748 Stereocaulon octomerellum
KF644367 Stereocaulon nigrum
DQ396955 Stereocaulon japonicum
DQ396948 Stereocaulon exutum
DQ396969 Stereocaulon pendulum
DQ396950 Stereocaulon exutum
KY660026 Stereocaulon dendroides
DQ396947 Stereocaulon depreaultii
KP324750 Stereocaulon verruculigerum
DQ396945 Stereocaulon japonicum
HQ650669 Frutidella pullata
MW257114 Pulvinora pringlei
MW257117 Pulvinora subcavicola
OQ650067 Lecanora argentata
DQ534469 Lecanora epibryon
OQ717426 Lecanora allophana
MK620141 Lecidella stigmatea
OQ557090 Lecidella stigmatea
JN873884 Lecidella greenii
JN873895 Lecidella siplei
JN873894 Lecidella patavina
KT453757 Lecidella enteroleucella
JN873903 Lecidella wulfenii
PQ099899 Lecidella elaeochroma
OQ534854 Lecidella drakensis
Stereocaulaceae
85.4 / 95
99.3 / 100
91.3 / 98
90.7 / 98
90 / 99
99.3 / 100
91.7 / 98
94.1 / 100
95 / 100
95.4 / 100
93.1 / 100
86.9 / 100
100 / 100
93.8 / 97
91.2 / 100
82.4 / 97
91.6 / 100
93.3 / 99
99.9 / 100
82.4 / 100
86.1 / 96
98 / 100
97.9 / 100
95.1 / 100
97.5 / 100
93.4 / 98
98.9 / 100
99.5 / 100
95.3 / 100
99.8 / 100
96.1 / 100
99.6 / 100
97 / 100

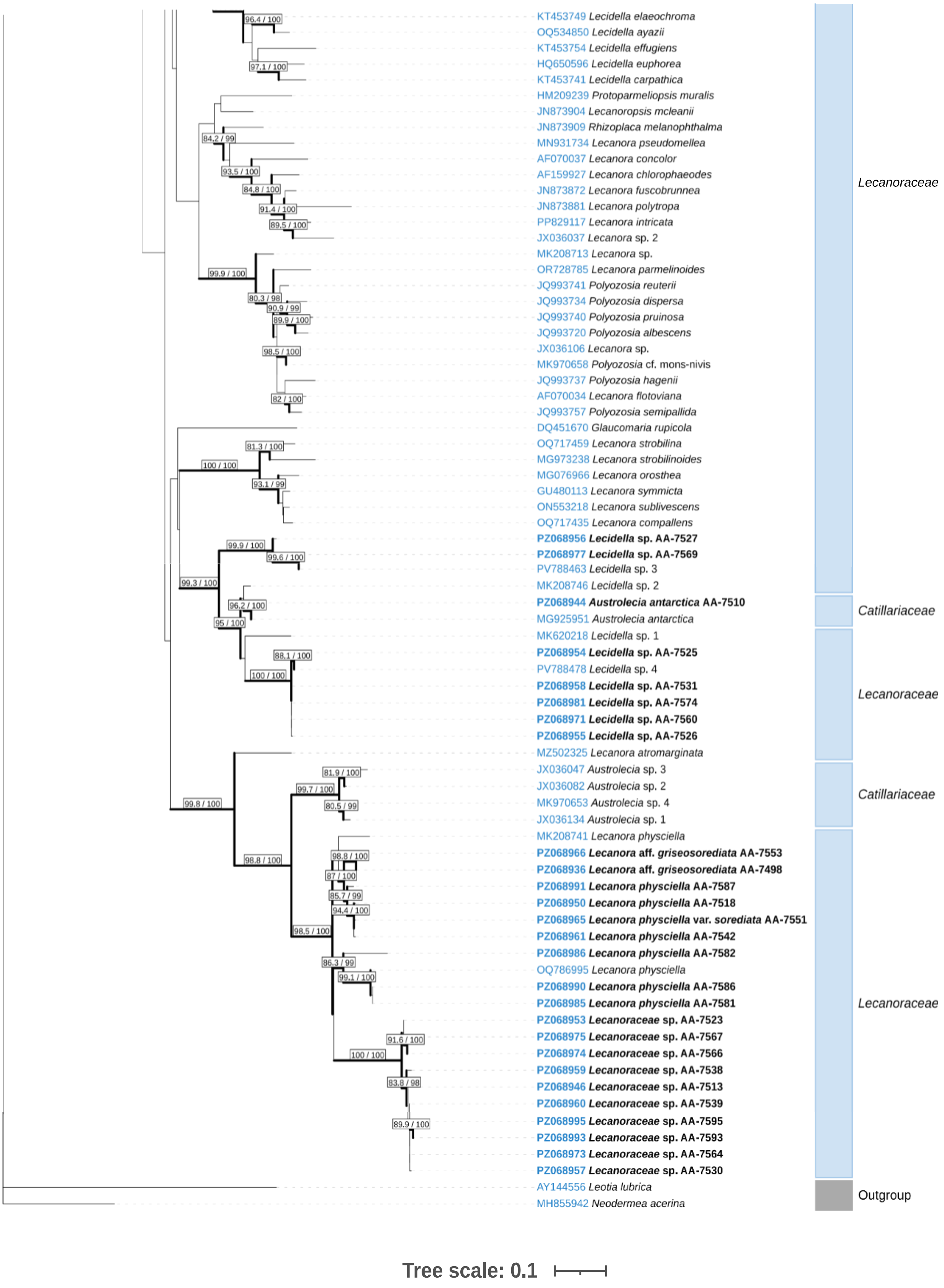
96.4 / 100
97.1 / 100
84.2 / 99
93.5 / 100
84.8 / 100
91.4 / 100
89.5 / 100
99.9 / 100
80.3 / 98
90.9 / 99
89.9 / 100
98.5 / 100
82 / 100
81.3 / 100
100 / 100
93.1 / 99
99.9 / 100
99.6 / 100
99.3 / 100
96.2 / 100
95 / 100
88.1 / 100
100 / 100
99.8 / 100
81.9 / 100
99.7 / 100
80.5 / 99
98.8 / 100
98.8 / 100
87 / 100
85.7 / 99
94.4 / 100
98.5 / 100
86.3 / 99
99.1 / 100
91.6 / 100
100 / 100
83.8 / 98
89.9 / 100
KT453749 Lecidella elaeochroma
OQ534850 Lecidella ayazii
KT453754 Lecidella effugiens
HQ650596 Lecidella euphorea
KT453741 Lecidella carpathica
HM209239 Protoparmeliopsis muralis
JN873904 Lecanoropsis mcleanii
JN873909 Rhizoplaca melanophthalma
MN931734 Lecanora pseudomellea
AF070037 Lecanora concolor
AF159927 Lecanora chlorophaeodes
JN873872 Lecanora fuscobrunnea
JN873881 Lecanora polytropa
PP829117 Lecanora intricata
JX036037 Lecanora sp. 2
MK208713 Lecanora sp.
OR728785 Lecanora parmelinoides
JQ993741 Polyozosia reuterii
JQ993734 Polyozosia dispersa
JQ993740 Polyozosia pruinosa
JQ993720 Polyozosia albescens
JX036106 Lecanora sp.
MK970658 Polyozosia cf. mons-nivis
JQ993737 Polyozosia hagenii
AF070034 Lecanora flotoviana
JQ993757 Polyozosia semipallida
DQ451670 Glaucomaria rupicola
OQ717459 Lecanora strobilina
MG973238 Lecanora strobilinoides
MG076966 Lecanora orosthea
GU480113 Lecanora symmicta
ON553218 Lecanora sublivescens
OQ717435 Lecanora compallens
PZ068956 Lecidella sp. AA-7527
PZ068977 Lecidella sp. AA-7569
PV788463 Lecidella sp. 3
MK208746 Lecidella sp. 2
PZ068944 Austrolecia antarctica AA-7510
MG925951 Austrolecia antarctica
MK620218 Lecidella sp. 1
PZ068954 Lecidella sp. AA-7525
PV788478 Lecidella sp. 4
PZ068958 Lecidella sp. AA-7531
PZ068981 Lecidella sp. AA-7574
PZ068971 Lecidella sp. AA-7560
PZ068955 Lecidella sp. AA-7526
MZ502325 Lecanora atromarginata
JX036047 Austrolecia sp. 3
JX036082 Austrolecia sp. 2
MK970653 Austrolecia sp. 4
JX036134 Austrolecia sp. 1
MK208741 Lecanora physciella
PZ068966 Lecanora aff. griseosorediata AA-7553
PZ068936 Lecanora aff. griseosorediata AA-7498
PZ068991 Lecanora physciella AA-7587
PZ068950 Lecanora physciella AA-7518
PZ068965 Lecanora physciella var. sorediata AA-7551
PZ068961 Lecanora physciella AA-7542
PZ068986 Lecanora physciella AA-7582
OQ786995 Lecanora physciella
PZ068990 Lecanora physciella AA-7586
PZ068985 Lecanora physciella AA-7581
PZ068953 Lecanoraceae sp. AA-7523
PZ068975 Lecanoraceae sp. AA-7567
PZ068974 Lecanoraceae sp. AA-7566
PZ068959 Lecanoraceae sp. AA-7538
PZ068946 Lecanoraceae sp. AA-7513
PZ068960 Lecanoraceae sp. AA-7539
PZ068995 Lecanoraceae sp. AA-7595
PZ068993 Lecanoraceae sp. AA-7593
PZ068973 Lecanoraceae sp. AA-7564
PZ068957 Lecanoraceae sp. AA-7530
AY144556 Leotia lubrica
MH855942 Neodermea acerina
Lecanoraceae
Catillariaceae
Lecanoraceae
Catillariaceae
Lecanoraceae
Outgroup
Tree scale: 0.1

Figure S3. Bar plot showing the number of taxa per rank in both nunatak and non-nunatak environments, relative to the total recorded taxa from each area (Non-Nunatak: n = 559, Nunatak: n = 255).

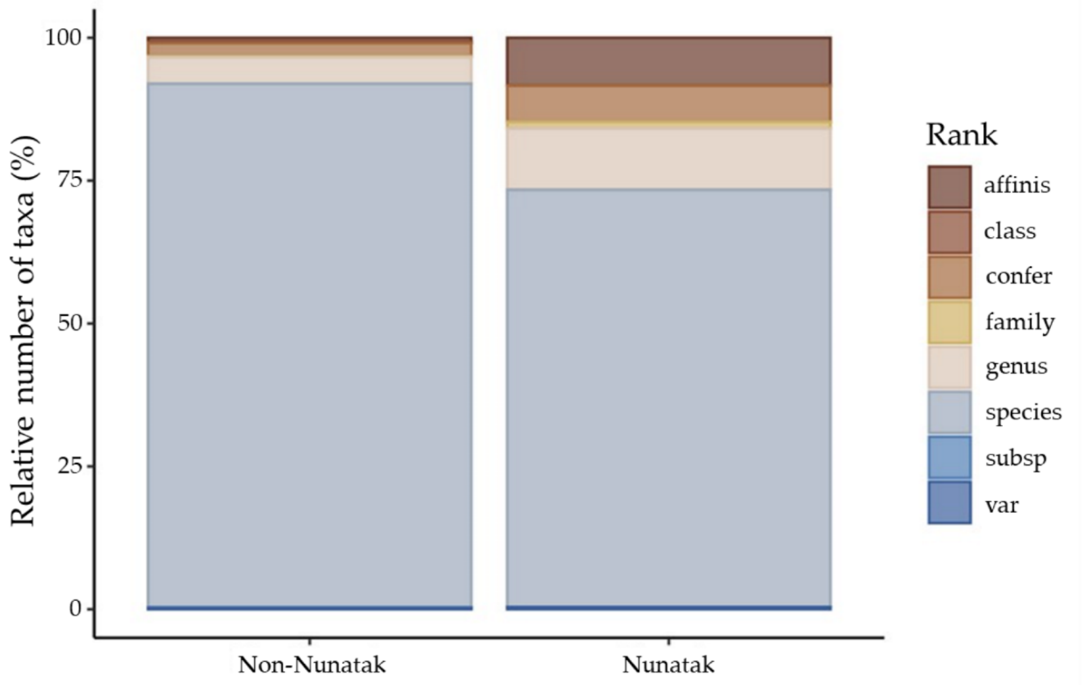

Figure S4. Bar plot depicting the biogeographic origin of species, subspecies, and varieties in nunataks from the continental (green) and maritime regions (yellow).

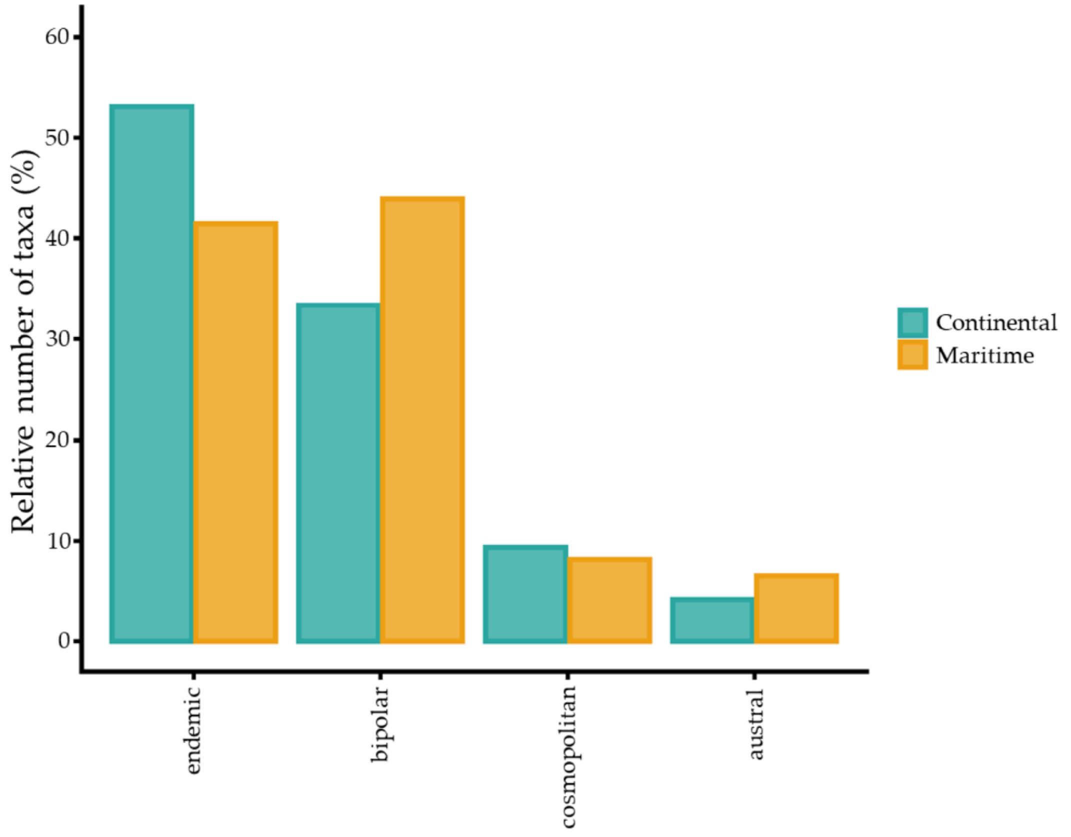

Figure S5. Bar plot depicting the biogeographic origin of species and varieties in the Hurd Peninsula non-nunatak (black) and nunatak areas (red). The nunataks considered include the four surveyed in the present study (Nunatak del Castillo, Napier Peak, Cerro Mirador, and MacGregor Peak) as well as Moores Peak.

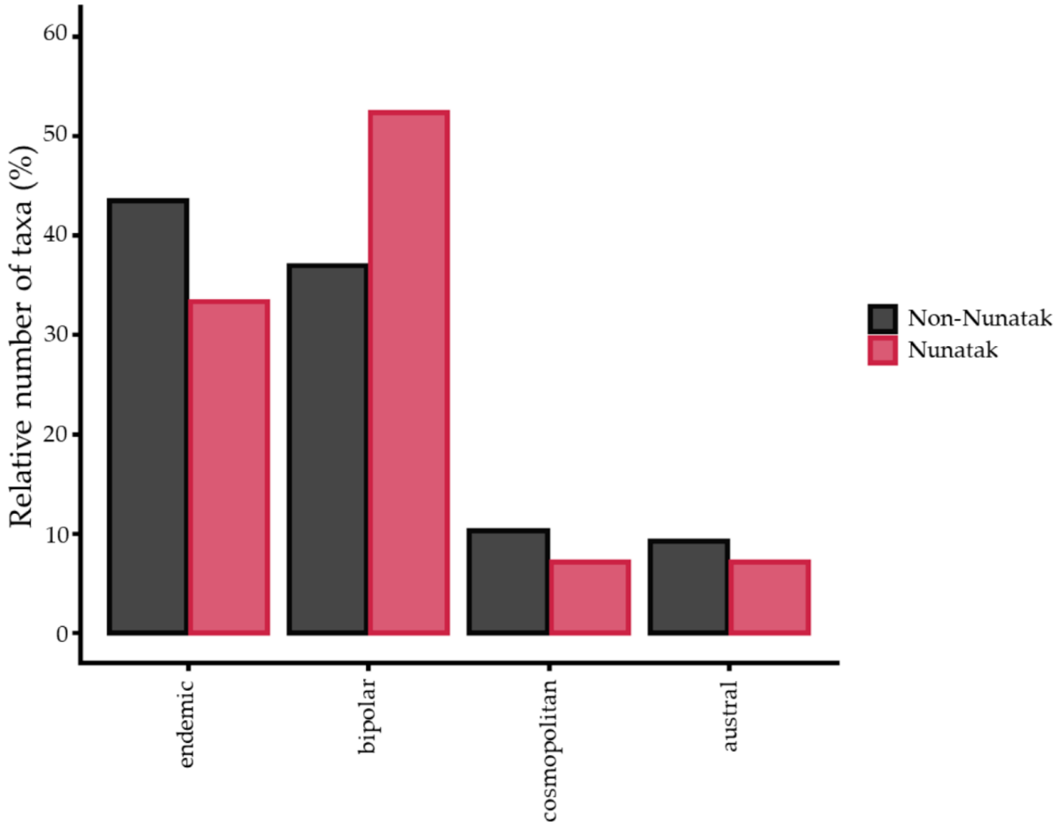

Figure S6. Statistical parsimony networks for the following species based on the nrITS marker: *Acarospora gwynii*, *Austrolecia antarctica*, *Lecanora fuscobrunnea*, *L. physciella*, *Lecidea cancriformis*, *Lecidella siplei*, *Pseudephebe minuscula*, and *Usnea lambii*. Samples are grouped by region and habitat (see Table S5): non-nunatak environments in continental Antarctica (light blue), nunatak environments in continental Antarctica (dark blue), non-nunatak environments in maritime Antarctica (light orange), and nunatak environments in maritime Antarctica (dark orange). The number of samples sharing each haplotype is indicated in italics below the corresponding haplotype label. Within each haplotype network, circle size is proportional to haplotype frequency. Circle sizes are not directly comparable among networks due to differences in sequence abundance across species. Mutational steps are represented by hatch marks along the connections between haplotypes, with each mark corresponding to a single nucleotide difference.

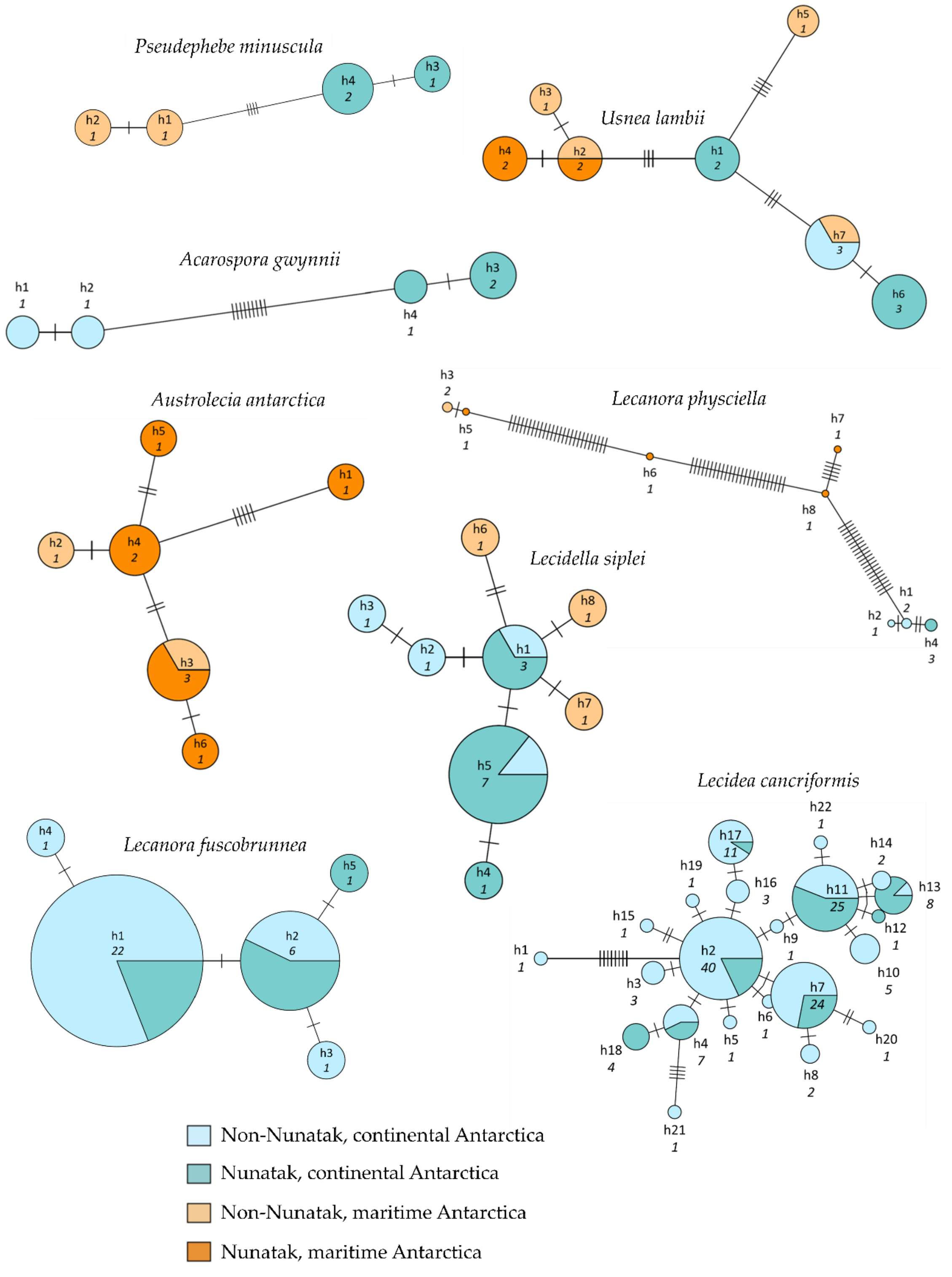
Pseudephebe minuscula
Usnea lambii
Acarospora gwynnii
Austrolecia antarctica
Lecanora physciella
Lecidella siplei
Lecidea cancriformis
Lecanora fuscobrunnea
Non-Nunatak, continental Antarctica
Nunatak, continental Antarctica
Non-Nunatak, maritime Antarctica
Nunatak, maritime Antarctica
